# Impact of Governmental interventions on epidemic progression and workplace activity during the COVID-19 outbreak

**DOI:** 10.1038/s41598-021-01276-5

**Published:** 2021-11-09

**Authors:** Sumit Kumar Ram, Didier Sornette

**Affiliations:** 1 Department of Management Technology and Economics, ETH Zurich, Zurich, Switzerland; 2 Department of Earth Sciences, ETH Zurich, Zurich, Switzerland; 3 Department of Physics, ETH Zurich, Zurich, Switzerland; 4 Swiss Finance Institute c/o University of Geneva, Geneva, Switzerland; 5 Institute of Risk Analysis, Prediction and Management (Risks-X), Academy for Advanced Interdisciplinary Studies, Southern University of Science and Technology (SUSTech), Shenzhen, 518055 China; 6 Tokyo Tech World Research Hub Initiative, Institute of Innovative Research, Tokyo Institute of Technology, Tokyo, Japan; 7 Connection Science, Massachusetts Institute of Technology, Cambridge, MA USA

**Keywords:** Statistical physics, thermodynamics and nonlinear dynamics, Infectious diseases

## Abstract

In the first quarter of 2020, the COVID-19 pandemic brought the world to a state of paralysis. During this period, humanity saw by far the largest organized travel restrictions and unprecedented efforts and global coordination to contain the spread of the SARS-CoV-2 virus. Using large scale human mobility and fine grained epidemic incidence data, we develop a framework to understand and quantify the effectiveness of the interventions implemented by various countries to control epidemic growth. Our analysis reveals the importance of timing and implementation of strategic policy in controlling the epidemic. We also unearth significant spatial diffusion of the epidemic before and during the lockdown measures in several countries, casting doubt on the effectiveness or on the implementation quality of the proposed Governmental policies.

## Introduction

The pandemic due to the SARS-CoV-2 virus^1^ impacted the world population, health care system and economies in 2020^2^. Since its identification in December 2019 in Wuhan, China, this novel coronavirus disease (COVID-19) continued to spread in China in Jan.-Feb. 2020. The epidemic was detected in Italy in the second half of February and progressively diffused in the rest of the world. It was declared a global pandemic on March 11, 2020 by the World Health Organization (WHO)^3^. We all witnessed and experienced a series of interventions with various levels of confinement measures^4^ in different countries and regions, aimed at decreasing the effective reproduction number $R_t$ and controlling the epidemics. Yet, how to assess the impact of the intervention policies to contain the epidemic remains an important issue. In this article, we present a framework to understand and quantify the effectiveness of the interventions implemented by various countries to control epidemics.

As of the last update of this article (25th August 2021), 20–21 months after the virus was originally described, more than 213 million reported SARS-CoV-2 infections have occurred worldwide (from more than 210 countries). COVID-19 has been linked to almost 4.46 million deaths^5^. Over the multiple waves, this epidemic has posed and is still posing a significant threat to human physical and mental health^6^, and it has had a significant influence on daily life, with psychosocial consequences^6^ on a global scale. Further, the infection dynamics has revealed the effect of hidden environmental factors like air pollution on the severity of the spread of the contagion^7,8^.

Our starting hypothesis is that the emergence of infected cases, the various types of illnesses caused by infection, and the death rates reflect the interaction between the biological and epidemiological properties of this new SARS-CoV-2 virus and the political, cultural, sociological, and governance characteristics of various nations and human communities. By combining mobility data, epidemiological data and clinical data across ten countries, we develop a modelling framework to quantify the effectiveness of the interventions implemented by various countries to control epidemic growth. This shock provides a real-life natural experiment to falsify the effectiveness of different organisations and interventions and policies, with the goal of informing and guiding future plans against potential second and third waves of the epidemics as well as future outbreaks in general.

First, we quantify precisely how interventions in the form of mobility restrictions and social distancing have had significant impacts on controlling the development of epidemics in many countries, as measured by the decrease in reproduction number $R_t$ and the extent of curbing the increase in infected cases. We also document a surprisingly large heterogeneity in the reduction of $R_t$ across regions within a given country and also across countries. Further, we observe non-monotonic and fluctuating time dependence of $R_t$ in different regions.

The surprising result is that, in many regions where the epidemic was not visible, the interventions led to a significant transient increase in reproduction numbers $R_t$, in contradiction with the short-term objective of lockdown measures. For instance, in several regions of France and Italy, lockdown led to an obvious strong collapse in mobility, accompanied by an increase in $R_t$. We note that this is not due to the inherent delay in case detection due to the incubation period, symptoms’ onset and testing. We interpret this phenomenon as a result of preemptive large movements of people to relocate before the strict lockdown implementation, hence promoting new contagions and epicenters for the epidemics to mature for a while after the lockdown. Another plausible mechanism, which underlies the Japanese policy, is the effect of closed spaces and close-contact settings within confined households.

We also quantify how the timing of the lockdown determined the trajectory of the epidemic by estimating the spatial correlation across regions within a country of the total increase of infected cases after the lockdown. In a number of countries, the epidemic started much before the lockdown date and was developing silently, as revealed by the strong spatial diffusion of the epidemic after the lockdown. In other countries, the epidemic was better contained by intervention measures.

Our analysis overall suggests that the interventions may have not been optimal and that there are probably better alternatives to complete lockdowns. Our study reveals the importance of timing and targeting of interventions as a likely better strategy compared with undifferentiated lockdown. We observe belated intervention at the regional and local levels and hasty global lockdowns, which, in a number of regions, result in a disappointing reduction in the reproduction numbers and a curbing of the increase in new cases compared to other comparable countries.

## Materials and methods

### Data

#### Clinical data

##### Incubation period and confirmation period

To calculate various epidemiological parameters, we use the individual-level data^9,10^ on COVID-19 epidemic which are geo-coded and includes symptoms, key dates (date of onset, admission, and confirmation), and travel history. The dataset is curated from different sources, including official government sources (official websites of Ministries of Health or Provincial Public Health Commissions), peer-reviewed scientific articles, online reports, and news websites. From this database, we selected 216 individuals, who got clinical confirmation of covid-19 and were symptomatic as well as traveled to suspected places of exposure. We have the exact dates of travel, onset of symptoms, and clinical confirmation for these 216 individuals.

##### *Serial interval*

 We use the infector-infectee pairs dataset that has been collected from publicly available information published in research articles and quoted from official reports of outbreak investigations^11^. We use 28 probable pairs for the estimation of generation time. For these 28 pairs of individuals, the date of illness onset for the pairs of individuals is defined as the date on which a symptom relevant to COVID-19 infection appeared and is determined by the reporting governmental body.

#### Epidemiological data

We collect the daily reported cases at the first administrative level divisions for a list of countries from various sources. The primary sources of information for the datasets are often local news agencies, government reports, WHO reports, and various medical communities. Table 1 gives a list of countries with their starting date of governmental interventions. There is no lockdown in Japan like in other countries. However, prime minister Abe on 7 April, proclaimed a state of emergency. This was the first emergency declaration in Japan and we consider this date as the starting date of the intervention.

**Table 1:** Starting dates of Governmental interventions (includes the lockdown) for controlling the outbreak. (*Source*: https://en.wikipedia.org/wiki/COVID-19_pandemic_lockdowns)

Country	China	Italy	Spain	Switzerland	France	Canada	United States	Germany	India	Japan
Intervention date	2020-01-23	2020-03-09	2020-03-14	2020-03-16	2020-03-17	2020-03-18	2020-03-19	2020-03-20	2020-03-25	2020-04-07

#### Mobility data

We use the aggregated anonymised community mobility dataset that has been collected through the Google Maps app^12^, to help understand what has changed in response to the government policies aimed at flattening the curve of the COVID-19 pandemic. The dataset is anononymised to ensure that no personal data, including an individual’s location, movement, or contacts, can be derived from the resulting metrics. The anonymization process for the data includes differential privacy^13^, with intentionally added random noise to metrics in a way that maintains both users’ privacy and the overall accuracy of the aggregated data^14^. The dataset contains the percentage changes in the anonymized mobility metrics of Google users from a baseline based on the historical part. The dataset is prepared by counting and accordingly standardizing the number of unique users before and after the interventions, who visited a public place in a given category on a given day at a different granularity level. There are seven different categories derived from the data: retail, recreation, eateries (retail & recreation), groceries, pharmacies, transit, and parks.

#### Map data

We collected the geojson files for the first-level administrative divisions of the list of countries containing the names of the regions and the geometry from a number of openly accessible github repositories.

### Models and methodology

For our analysis, we select a time span that starts 10 days before the implementation of Governmental interventions—for controlling the outbreak, often through mass lockdowns—and ends 30 days following the starting date of Governmental interventions.

#### Epidemiological estimations

The time between exposure and onset of symptoms is defined as the incubation period. We use a Bayesian framework to model the day of onset of symptoms following the date of exposure to the virus and the day of clinical confirmation of the virus following the date of exposure. Using the key dates for the selected 216 individuals^9,10^, we estimate the important epidemiological parameters like incubation period, confirmation period (the number of days it take to get the clinical confirmation about the virus following the date of exposure). We use the logistic model

1 $$\begin{aligned} P(conf./sympt. | t)=\frac{1}{1+e^{\beta t+\alpha }} \end{aligned}$$

to estimate the cumulative probability distribution of individuals developing symptoms or getting the clinical confirmation on the $day=t$ that they are infected, conditional on the fact that the individual was exposed to the virus on $day=0$ and will be eventually symptomatic and will get a clinical *+ve* confirmation result. With the help of Metropolis-Hastings algorithm^15^,—a Markov Chain Monte Carlo (MCMC) method^16^– we sample the model parameters ($\alpha ,\beta$), from a standard normal prior and train our model on the dataset to estimate the posterior probability distribution of developing symptoms or getting the clinical confirmation on the $day=t$, following the day of exposure on $day=0$.

#### Estimation of the time-dependent effective reproduction number* R*_*t*_

The principal epidemiological variable characterizing a disease’s transmission potential is the basic reproduction number, $R_0$, which is characterized as the estimated number of secondary cases caused by a typical primary case, in an entirely susceptible population. When an infection is spreading across a population, working with the effectively reproductive number $R_t$, which is defined as the actual average number of secondary cases per primary case, is often more convenient. $R_t$ is normally smaller than $R_0$, which reflects the impact of epidemic controls and the decline of susceptible individuals. The value of $R_t$ is comparable to the branching ratio in the Hawkes process^17,18^, where $R_t>1$ can lead to explosive growth of the epidemic and an ever-increasing number of new cases, while $R_t<1$ leads to the eventual demise of the growth process. Provided the branching structure describing who infected whom is determined, it becomes trivial to estimate $R_t$. However, this information is not generally available and the estimation of $R_t$ then becomes tricky. Nevertheless, recent advances in epidemiology have made it possible to estimate the time evolution of the effective reproduction number ($R_t$) through the observed epidemic curve in a geographical region^19,20^. We use the method introduced by^20^, which uses the daily number of confirmed cases and a model described below for the generation time (which is one of the key parameters dictating the severity of epidemic growth) to estimate the temporal evolution of the effective reproduction number ($R_t$) with the help of a Sequential Bayesian estimation approach.

The generation time is defined for source-recipient transmission pairs as the time between the infection of the source and the infection of the recipient. Because the time of infection is generally not known, the generation time is often approximated by the serial interval, which is defined as the time between the onset of symptoms of the source and the onset of symptoms of the recipient. For the present case, we use the data for the serial intervals from^11^, which has been constructed using publicly available data. We calibrate the data against three models, i.e., Weibull, Log-normal, and Gamma distributions, to find the best possible approximation model from which the generation time is determined.

We use a probabilistic contagion model with inhomogenous source terms to explain the progression of the COVID-19 epidemic^20^. We consider both human to human transmission and infections from the reservoir (contaminated surfaces) to explain the epidemic growth. Denoting *S*(*t*) and *N*(*t*) as the average number of susceptibles and total population at time *t* and $\beta$ and $\gamma ^{-1}$ as the contact rate and the infectious period, respectively,

2 $$R_0 = \frac{\beta }{\gamma }\quad \mathrm{and}\quad R_t=\frac{S(t)}{N(t)}\times R_0. $$

According to this model, if we denote the number of new infections from the reservoir between *t* and $t+\tau$ by $\Delta B(t)$ and the number of new cases within this period by $\Delta T(t+\tau )$, then the stochastic discrete variable $\Delta T(t+\tau )$ is generated by a probability distribution with the average number of cases given by

3 $$\begin{aligned} \begin{aligned} \Delta T(t+\tau ) \sim P\left\{ \lambda \right\} \end{aligned} \end{aligned}$$

with

4 $$\begin{aligned} \begin{aligned} \lambda = \Delta B(t+\tau )+b\left( R_{t}\right) \left( \Delta T(t)-\Delta B(t)+\tau \gamma R_{t} \Delta B(t)\right) \end{aligned} \end{aligned}$$

where $P\{ \lambda \}$ denotes a discrete probability distribution with a mean $\lambda$. In eq. (4), $b(R_t)$ can be expressed as

5 $$\begin{aligned} b(R_t) =exp\left[ \tau \gamma (R_t-1)\right] ~, \end{aligned}$$

which is the slope of the tangent at the origin of case trajectories from the epidemic time delay plot ($\Delta (t)$ vs $\Delta (t-\tau )$) of surveillance data. More details about the derivation of the model can be found in the supplementary material or in^20^.

We use a Bayesian framework to estimate the full probability distribution for the effective reproduction number $R_t$, conditional on the time series for new cases. The probability distribution of $R_t$, compatible with the observed temporal data stream, is given by

6 $$\begin{aligned} P[R_t | \Delta T(t+\tau ) \leftarrow \Delta T(t)]=\frac{P[\Delta T(t+\tau ) \leftarrow \Delta T(t) | R_t] P[R_t]}{P[\Delta T(t+\tau ) \leftarrow \Delta T(t)]}~, \end{aligned}$$

where $P[R_t]$ is the prior distribution and $P[\Delta T(t+\tau ) \leftarrow \Delta T(t)]$ is independent of $R_t$, and is a normalization parameter. From successive applications of Bayes’ theorem, a sequential estimation scheme, that uses streaming epidemiological observations performed in real time, can be constructed using the posterior distribution for $R_t$, at time *t* as the prior in the next estimation step at time $t+\tau$, leading to an update scheme via iteration of eq. (6). The resulting probability distribution for $R_t$ includes information on all observations up to time *t*, and thus is a robust estimator of the effective reproduction number compared to the estimation by only considering the cases between *t* and $t+\tau$. Any changes in $R_t$ over time result from the assimilation of each new data point, leading to an updated estimate of $R_t$. This in turn allows us to use the estimation procedure as an anomaly detection tool.

#### Estimation of the impact of Governmental intervention

We use the estimated time evolving effective reproduction number and the time evolving mobility metric to study the impact of various governmental interventions to contain the spread of COVID-19. We use the framework proposed by^21^ to infer the impact on the epidemic progression as well as the human mobility because of various government interventions. The method uses a diffusion-regression state-space model that predicts the counterfactual evolution of effective reproduction number $R_t$, as well as the mobility metric in a synthetic control that would have occurred, had no intervention taken place. The model is successful in inferring the temporal evolution of attributable impact, and flexibly accommodates multiple sources of variation, including local trends, seasonality, and the time-varying influence of contemporaneous covariates by incorporating empirical priors on the parameters into a fully Bayesian framework. Using the MCMC for posterior inference, we estimate the most likely counter-factual evolution of the effective reproduction number and the mobility metric during the first 30 days of the interventions.

According to the model, the generalized Bayesian Structural Time Series—state-space models for time-series data—can be expressed by

7 $$\begin{aligned} y_t= & {} Z^T_t\alpha _t + \beta X_t + G_t\epsilon _t~, \end{aligned}$$

8 $$\begin{aligned} \alpha _{t+1}= & {} T_t\alpha _t + H_t\eta _t~, \end{aligned}$$

where $\epsilon _t \sim {\mathcal {N}}(0, \sigma _t^2)$ and $\eta _t \sim {\mathcal {N}}(0, Q_t)$ are independent of all other unknowns, $\alpha _t$ is referred to as a “state” of the series, and $y_t$ is a linear combination of the states plus a linear regression with the *covariates* *X*. Eq. 8 is the state equation governing the evolution of the state vector $\alpha _t$ through time, whereas $y_t$ is a scalar observation. It is possible to model several distinct behaviors for the time series (including *ARMA* or *ARIMA*) by varying the matrices $Z_t^T$, $T_t$, $G_t$, $Q_t$ and $H_t$.

Here, we simplify (8) by taking $T_t= H_t=1$, so that

9 $$\begin{aligned} \alpha _{t+1} = \alpha _t + \eta _{\mu , t} \end{aligned}$$

It is a simple random walk, also referred to as the “local level” component. This random walk component embodies the increasing uncertainty of observations as time passes. In (7), we take $Z^T_t=G_t=1$ but augment the equation by accounting for the possible presence of seasonal components embodied into $\gamma _t$ described below. This allows us to reduce expression (7) into

10 $$\begin{aligned} y_t = \alpha _t + \gamma _t + \beta X_t + \epsilon _t~. \end{aligned}$$

The seasonal components in eq. (10)) can be expressed as

11 $$\begin{aligned} \begin{aligned} \upgamma _t&= \sum _{j=1}^h \upgamma _{j, t} \\ \upgamma _{j, t+1}&= \upgamma _{j, t}\cos (\lambda _j) + \upgamma ^{*}_{j, t}\sin (\lambda _j) + \omega _{j,t} \\ \upgamma ^{*}_{j, t+1}&= -\upgamma ^{(1)}_{j, t}\sin (\lambda _j) + \upgamma ^{*}_{j, t}\cos (\lambda _j) + \omega ^{*}_{j, t}, \\ \omega ^{*}_{j, t}, \omega _{j, t}&\sim N(0, \sigma _{\omega ^2}) \\ \lambda _j&= \frac{2 \pi j}{s} \end{aligned} \end{aligned}$$

The linear dependence on the covariates $\beta X_t$ in eq. (10) further helps to explain the observed data. The better this component contributes to the prediction task, the lower the local level component $\mu _t$ should be. Finally, $\epsilon _t \sim {\mathcal {N}}(0, \sigma _t^2)$ models the noise associated with measuring $y_t$.

In order to estimate the impact of government interventions, we follow the following methodology. During the training period, we sampled the model parameters and the state vector using the Gibbs sampler—a MCMC framework—against the observed data. For the estimation of the impact on the mobility metric, the training period extends over the first 10 days of our selected dataset, i.e, from 10 days prior to the start of government intervention to the start of the intervention. For the estimation of the impact on the effective reproduction number, the training period extends over the first 20 days of our selected dataset, i.e, from 10 days prior and 10 days following the start of government intervention. We select this span of time since, for almost 50% of people, it takes at least 9.92 days following the date of exposure to get clinical confirmation of the viral infection (see Fig. 10 for the Hessen state in Germany). Hence, on average, there is a delay of around 10 days between the date of exposure and the date of confirmation, and there will not be any immediate effect on the daily confirmed cases as a result of the intervention. Thus, we define the estimated *effective intervention date* as being equal to the exact intervention date plus $9.92 \approx 10$ days. We then use the posterior simulations to simulate the posterior predictive distribution over the counterfactual time series given the observed pre-intervention activity during the training. We use the following 30 days for the mobility metric and the following 20 days for the effective reproduction number for this purpose.

Finally, we use the posterior predictive samples to compute the posterior distribution of the point wise impact, i.e, $\phi _t= y_t - {\tilde{y}}_t$, where $y_t$ is the observed quantity and ${\tilde{y}}_t$ is the counterfactual prediction assuming no intervention. We define the absolute impact of the intervention as the expected value of the point wise impact, i.e, $I=\langle \phi _t \rangle _t$. We used 30 days and 20 days of posterior predictive samples for the mobility metric and $R_t$ to estimate the point-wise impact as well as the absolute impact of the intervention. We also estimate the p-value of the observed absolute impact, which measures the probability of obtaining the impact by chance under the null of no intervention.

### Spatial auto-correlation measure (Moran’s I)

We define Moran's I which is a measure of spatial auto-correlation, as follows

12 $$\begin{aligned} I=\frac{N}{W} \frac{\sum _{j} \sum _{j} w_{i j}(\Delta S_{i}-\Delta {\bar{S}})\left( \Delta S_{j}-\Delta {\bar{S}}\right) }{\sum _{i}\left( \Delta S_{i}-\Delta {\bar{S}}\right) ^{2}} \end{aligned}$$

where *N* is the number of spatial units indexed by *i* and *j* in a given country; $\Delta S$ is the increase in the number of confirmed cases; $\Delta {\bar{S}}$ is the mean of $\Delta S$ over all the spatial units of the country; $w_{ij}$ is a matrix of spatial weights with zeroes on the diagonal (i.e., $w_{ii} = \textit{0}$); and *W* is the sum of all $w_{ij}$. We define *W* by giving a weight *1* if two regions are neighbors, and *0* otherwise. The “spatial lag” of $\Delta S$ for a region is defined as the weighted sum of its neighbors’ $\Delta S$.

## Results

**Figure 1:**
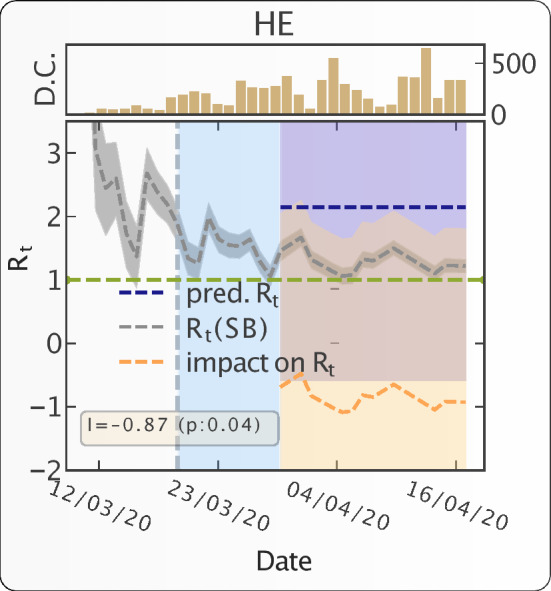
Progression of epidemic and impact of travel restrictions on epidemic growth in the Hessen state in Germany. The grey dashed line (with 95% CI band) represents the most likely estimation of $R_t$. The dotted vertical line represents the start date of intervention. The region between intervention date and effective intervention date—10 days following the intervention date—is marked with light blue color. It indicates the period during which people exposed to the virus prior to the intervention date would keep on appearing as the new confirmed cases. The counterfactual predicted $R_t$ is presented by the dashed blue line (with 95% CI band). The pointwise impact of the intervention is presented as the dashed orange line (with 95% CI band). The horizontal green line represents the critical value $R_t=1$. The inset figure shows the time evolution of number of daily confirmed cases (D.C.). We note down the absolute impact—average value of point wise impact—of the intervention along with the p-value in the yellow box. The p-value measures the probability of observing the impact by random chance.

We use a probabilistic contagion model with inhomogeneous source terms (e.g. transmission from contaminated surfaces, human to human transmission) to explain the temporal evolution of the epidemic because of COVID-19^20^. Using the daily number of confirmed cases and the generation time model (see Fig. (S1)), we estimate the time evolution of the effective reproduction number $R_t$—which is defined as the actual average number of secondary cases per primary case—with a Sequential Bayesian estimation. As a typical result of our analysis, Fig. 1 shows the time evolution of the effective reproduction number in the *Hessen state* in *Germany*.

### Impact of governmental interventions on mobility and* R*_*t*_

With the help of a diffusion-regression state-space model^21^ and MCMC posterior inference, we estimate the counterfactual evolution—that would have occurred had no intervention taken place—of the post intervention effective reproduction number. The comparison between the effective reproduction number and the predicted $R_t$, had no intervention taken place, allows us to quantify the effectiveness of intervention for the *Hessen state* in *Germany* shown in Fig. 1. We find an average reduction of $R_t$ of 0.87, and reject the null hypothesis with a p-value of 0.04 that this reduction could result from chance under counterfactual evolution without lockdown.

**Figure 2:**
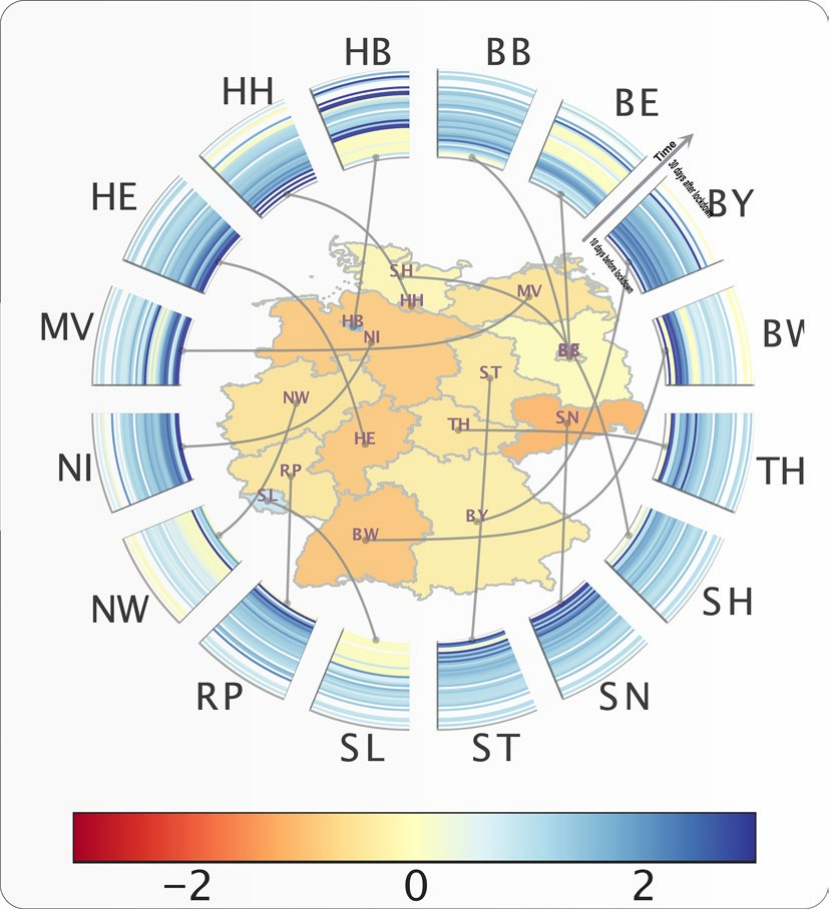
Time evolution of $R_t$ and absolute impact of travel restrictions on $R_t$ for different states in Germany. The color for each states (name in ISO 3166-2 code notation) represents the absolute impact (increase or decrease of $R_t$) due to travel restriction in that state. The radial wedges represent the time evolution of $R_t$ in the corresponding state and color of the strips represent $R_t$ on a particular day.

Figure 2 shows the map of the absolute impact on $R_t$ as a result of intervention by different states in Germany. The color for each state represents the average impact of the intervention on $R_t$, i.e., the magnitude increment or decrement of $R_t$ from the counterfactual predicted value without lockdown over the 30 days following lockdown. The radial wedges represent the temporal evolution of $R_t$ in the corresponding state and the color of the strips represents $R_t$ on a particular day over the 30 days following lockdown. This figure illustrates the significant heterogeneity in the results, as well as the important non-monotonicity in the dynamics of $R_t$.

**Figure 3:**
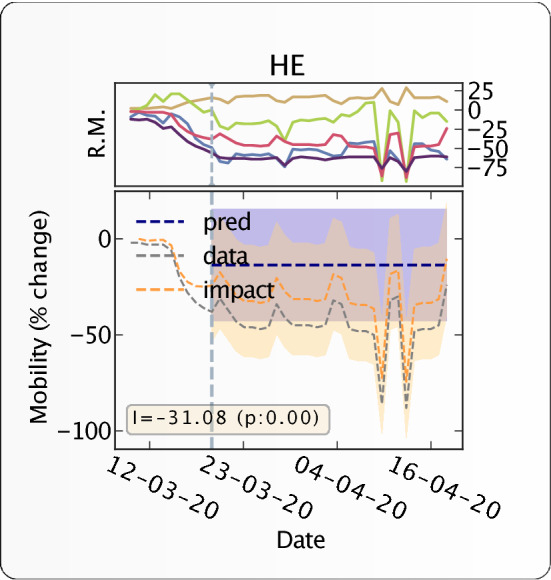
Countrywide time evolution of human mobility and impact of travel restrictions on human mobility for Germany. The top panel represents the time evolution of human mobility (% change from the baseline activity) in different mobility dimensions (yellow: home, red: work, blue: retail, green: grocery, violet: transit). Transit is a proxy for long-distance travel (it corresponds to petrol pumps/filling stations etc.). In the bottom panel, The y-axis represents % increase or % decrease of the average individual’s activity. the grey dashed line represents the % change of activity from the baseline (baseline is set to 0) in the workplace resulting from the intervention. The dotted vertical line represents the intervention date. The counterfactual predicted evolution of workplace activity, had no intervention taken place, is presented by the dashed blue horizontal line. The point-wise impact of the intervention on mobility is presented by the orange dashed line. The yellow box indicates the absolute impact of the intervention along with its p-value.

**Figure 4:**
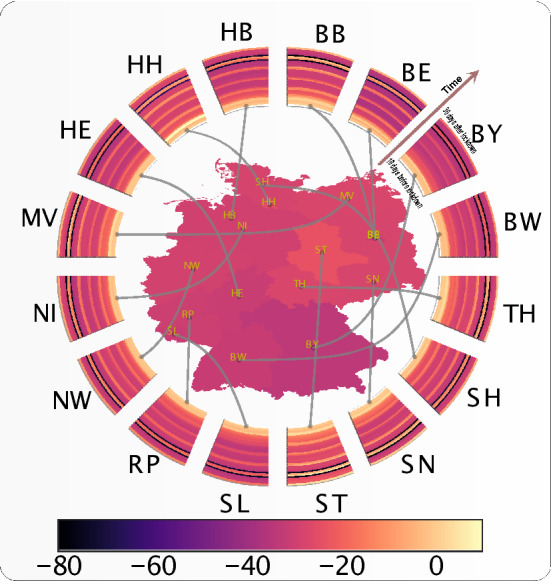
State-by-state time evolution of mobility and absolute impact of travel restrictions on mobility. The color of each state (name in ISO 3166-2 code notation) represents the absolute impact (increase or decrease of mobility) of interventions on workplace activity in that state. The radial wedges represent the time evolution of mobility in the corresponding state and color of the strips represent the mobility on a particular day. SL is Saarland, ST is Sachsen-Anhalt.

We then analysed the impact of government interventions on human mobility, illustrating the results for Germany. We are able to breakdown the impact of intervention into different mobility dimensions and quantify the level of reduction in mobility (see Fig. 3 for details). Figure 4 shows the absolute impact on workplace activity (in % change from baseline) in different states in Germany, resulting from the intervention. The radial wedges represent the temporal evolution of workplace activity in the corresponding state, and the color of the strips represents activity on a particular day. Contrary to the map of the absolute impact on $R_t$ (Fig. 2), the absolute impact of travel restrictions on mobility is much more homogenous across German states, and also consistent along the time axis.

**Figure 5:**
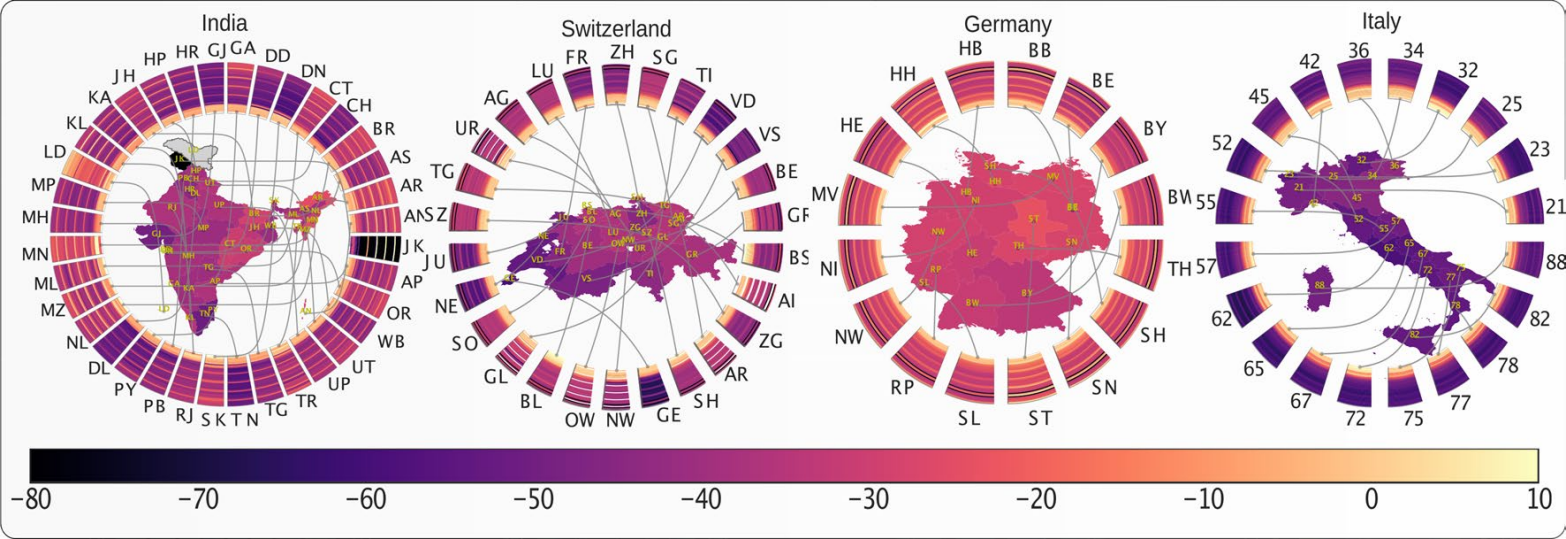
Time evolution of mobility and impact of travel restriction on mobility in four countries. Each subplot shows the time evolution of mobility and impact of travel restrictions on mobility of different top level administrative divisions of a country. The countries are, for left to right: India, Switzerland, Germany, Italy. The color of the regions on the map denoted by their ISO 3166-2 code represent the impact (increase or decrease of mobility) of travel restriction on that region. The radial connected wedges represent the time evolution of the mobility in the corresponding region or state for each country. The color of the strips in the wedges represent the mobility on a particular day.

**Figure 6:**
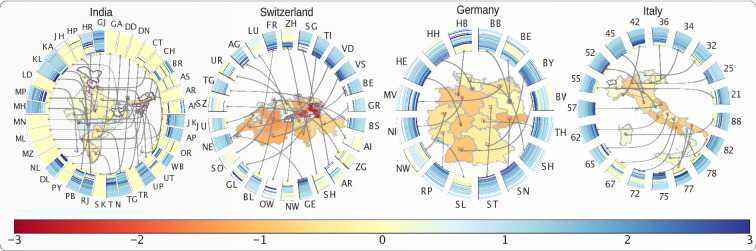
Time evolution of effective reproduction number ($R_t$) and absolute impact of travel restrictions on $R_t$ in four countries. Each subplot presents time evolution of $R_t$ and impact of interventions on $R_t$ for different top level administrative divisions of the country. The color of the regions (name in ISO 3166-2 code notation) represents the impact (increase or decrease of $R_t$) of interventions on that state. The radial wedges represent the time evolution of $R_t$ in the corresponding state, and color of the strips represent $R_t$ on a particular day. The countries are, from top left to bottom right: India, Switzerland, Germany,and Italy.

We then evaluate the impact of government interventions on $R_t$ as well as on works place activity for the top level administrative divisions in a number of countries. Figures 5 and S2 illustrate the impact of governmental interventions on workplace activity in a number of countries, and Figs. 6 and S3 (further expanded in Fig S16–S25) show the impact on $R_t$. Each subplot of Fig. 5 shows the time evolution of mobility and the impact of travel restrictions on mobility of different top level administrative divisions of a country. Each ring map represents a country and provides a visual representation of the time evolution of workplace activity as well as the impact of government interventions on workplace activity. Each subplot of Fig. 6 shows the time evolution of the effective reproduction number $R_t$ and the absolute impact of travel restrictions on $R_t$ in four countries. Each ring map represents a country and shows the time evolution of the effective reproduction number $R_t$ as well as the impact of government interventions on $R_t$.

Figures 5 and S2 (further expanded in Figs. S7–S15) show a rather homogeneous impact on workplace activities across the different regions of each of the four analysed countries. However, there is a large heterogeneity across different countries, e.g. Spain is the most affected country, while Japan is the least affected country in terms of workplace activity. In contrast, the impact on $R_t$ is quite heterogeneous across different regions within a country, notwithstanding similar levels of restriction, as illustrated in Fig. 6 by Kerala (KL) and Maharastra (MH) in India. Surprisingly, across a number of regions, a significant increment of $R_t$ is observed following the implementation of very strict lockdowns (e.g. Maharastra (MH) in India and Luzern (LU) in Switzerland.).

### Evolution of mobility and* R*_*t*_

**Figure 7:**
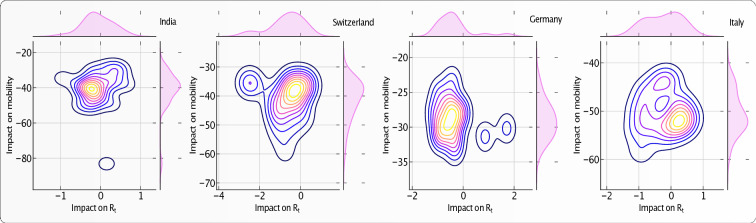
Impact of travel restrictions on workplace activity and on epidemic progression ($R_t$) in four countries. Each panel represents the bivariate kernel density estimation as a function of the absolute impact on workplace activity and impact on $R_t$ in the administrative divisions of each country. The bivariate distribution is constructed over the set of regions within each country. The top and right inset of each of the four plots represent the marginal distribution of the respective variables for each country. The countries are, from top left to bottom right: India, Switzerland, Germany, and Italy.

Figures 7 and S4 show the joint distribution (obtained by kernel density estimation) of the absolute impact resulting from intervention on workplace activity and on the $R_t$ in the administrative divisions of four different countries. In other words, Fig. 7 compares the strictness of governmental interventions, measured in terms of the impact on workplace activity, against the corresponding reduction/increment in effective reproduction number. There is no significant correlation between these two variables, suggesting that other variables are controlling the reduction in reproduction rates.

**Figure 8:**
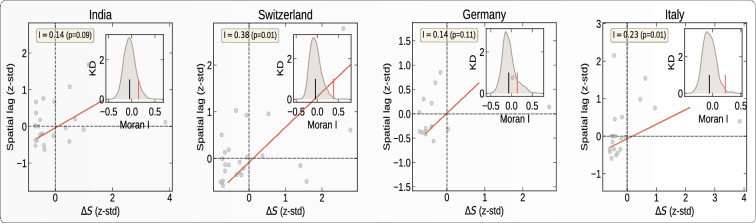
Spatial auto-correlation of the total increase $\Delta S$ in number of confirmed cases during the first 30 days of intervention in four countries. In each panel, the x-axis corresponds to the value of $\Delta S$ in a given region in a given country; the y-axis gives the average $\Delta S$ over the neighboring regions, called “spatial lag” in the caption along the y-axis. These two variables are *z-standardised* for better comparison. The inset in each panel represents the Kernel Density estimator for the distribution of the simulated *Moran’s I*. The black vertical line in the inset represents the expected *Moran’s I* from simulations with the null hypothesis of no spatial correlations. The red vertical line represents the value obtained from empirical data. *Moran’s I* along with its p-value is given in yellow box. The countries are, from top left to bottom right: India, Switzerland, Germany, and Italy.

Figures 8 and S5 represent the spatial correlation analysis (see “Spatial auto-correlation measure (Moran’s I)” section) of the total increase $\Delta S$ in the number of confirmed cases during the first 30 days of interventions. where *N* is the number of spatial units indexed by *i* and *j* in a given country; $\Delta S$ is the increase in the number of confirmed cases; $\Delta {\bar{S}}$ is the mean of $\Delta S$ over all the spatial units of the country; $w_{ij}$ is a matrix of spatial weights with zeroes on the diagonal (i.e., $w_{ii}$ = *0* ); and *W* is the sum of all $w_{ij}$. We define *W* by giving a weight *1* if two regions are neighbors, and *0* otherwise. The “spatial lag” of $\Delta S$ for a region is defined as the weighted sum of its neighbors’ $\Delta S$. The scatter plot in each panel of Fig. 8 shows the spatial lag as a function of its corresponding $\Delta S$ for different regions in each country. The inset gives the kernel density estimation of the simulated Moran’s I from the null model of no spatial correlation.

The slope of the scatter plot of $\Delta S$ against the spatial lag is known to converge to the *Moran’s I*^22^. We test the significance of the *Moran’s I* under the null hypothesis of no spatial auto-correlation and simulate *1000* realizations, by randomly shuffling the locations of the $\Delta S$. The spatial correlation analysis presented in Fig. 8 reveals a significant spatial correlation of $\Delta S$ in Italy, Switzerland, Japan, and the United States.

**Figure 9:**
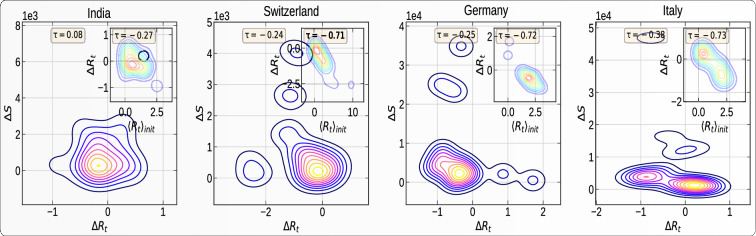
Joint distribution of the total number $\Delta S$ of confirmed cases within the first 30 days of intervention and of the absolute impact of intervention on the effective reproduction number ($\Delta R_t$) within this period. Each panel represents the Kernel Density Estimation for the total number of confirmed cases within the first *30* days of the intervention against the impact of interventions on effective reproduction number ($\Delta R_t$) in different administrative divisions of a country. The bivariate distribution is constructed over the set of regions within each country. The yellow box contains the Kendall $\tau$ correlation value for this joint distribution. The inset in each panel represents the variation of $\Delta R_t$ during the intervention against the average initial $R_t$ before the intervention. The yellow box gives the corresponding Kendalls $\tau$’s. The countries are, from top left to bottom right: India, Switzerland, Germany, and Italy.

In order to understand the effectiveness of lockdowns, we compared the number of confirmed cases against the impact on $R_t$ during the first 30 days of intervention. Figures 9 and S6 show the kernel estimation of the bivariate distribution of the total increase in number of cases and of the total impact on $R_t$ during the first 30 days of intervention in different regions of four countries. The inset in each panel represents the impact on $R_t$ during this period, (or $\Delta R_t$), against the average $R_t$ before the intervention (or $\langle R_t \rangle _{init}$) over the regions in each country.

The kernel density estimation of the bivariate distribution in Fig. 9 reveals a negative correlation between the above variables, showing a slowdown in epidemic growth notwithstanding the increase of $R_t$. This suggests the effectiveness of intervention measures in largely lowering the severity of epidemic. The places with increased $R_t$ only account for small growth of new cases. We also compare the average $R_t$ before the intervention (or $\langle R_t \rangle _{init}$) and impact of the intervention on $R_t$ (or $\Delta R_t$) to understand the effectiveness of intervention in reducing/increasing $R_t$ from its initial values. The inset panels of Fig. 9 unveil a negative correlation between these two variables, indicating a significantly large decrease of $R_t$ in the regions of larger initial $R_t$. Surprisingly, we also see the extension of the distribution to the second quadrant, revealing the fact that, in many places (e.g. Bremen, Germany), the epidemic started after the lockdown.

The tables S1 to S10 in the supplementary materials provide the detailed values of $R_t$ with positive and negative impacts along with the corresponding growth in the total number of confirmed cases as a result of intervention in the different regions of each country. The regions that are characterised by an increase in $R_t$ after lockdown are indicated in bold face.

## Discussion

**Figure 10:**
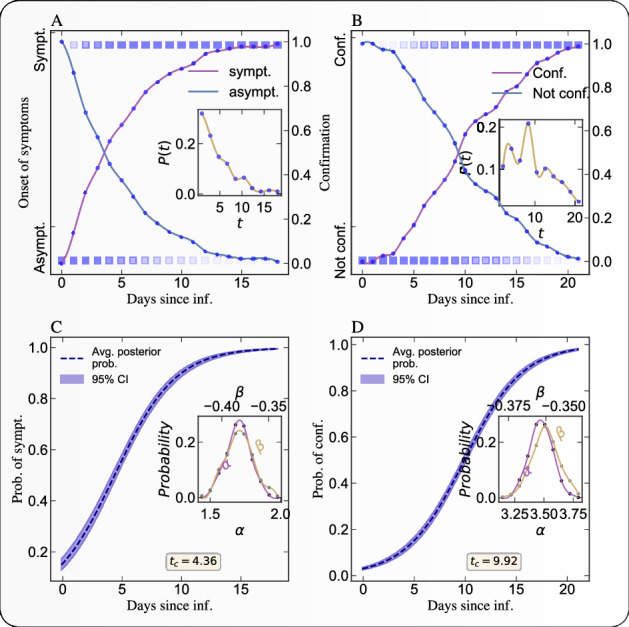
Bayesian inference of the incubation period and confirmation period. A) Empirical cumulative probability of developing a symptom on a particular day following the day of exposure to the virus. We use individual level clinical data^9,10^ to conduct these Bayesian estimations. The lighter color of the square represents low probability and the deep color represents high probability. The solid lines represent the fraction of people remaining asymptomatic (decreasing curve) or becoming symptomatic (increasing curve) on a given day. following exposure. The inset figure represents the empirical probability distribution of duration between the date of exposure and the date of onset of symptom or the clinical confirmation. B) Same as A) but for the probability of being confirmed. C) Estimation of the posterior probability distribution (with 95% confidence interval) of the incubation period (i.e., of getting the clinical confirmation on $day=t$, provided the individual is exposed to the virus on $day=0$). The dashed solid line represents the most likely posterior probability estimation and the light band represent the 95% confidence interval. The inset figure presents the distributions of the two estimated parameters in the logistic model (1). D) Same as C) for the probability distribution of confirmation period. The median value (and also mode) of the incubation period is 4.38 days. The median value (and also mode) $t_c$ of the confirmation period is 9.94 days.

We found it important to quantify the incubation and confirmation periods in order to assess the impact of policy interventions to contain the epidemics in different regions. This quantification, shown in Figs. 10 and S1 allows us to define a credible time interval over which to quantify the impact of interventions. With this, our analysis provides a framework to understand the effectiveness of the policies and interventions implemented by various countries to control the epidemic’s growth. The wealth of results presented in Figs. 1, 2, 3, 4, 5, 6, 7, 8 and 9 leads to three major observations: (1) interventions in the form of social distancing are found to have significant impacts; (2) large heterogeneity in the reduction of $R_t$ across different states within the same country; (3) transient increase in infection just after the lockdown measures. We discuss the above observations in detail in the following paragraphs.

### Observations

Overall, it is clear, and unsurprising, that interventions in the form of mobility restrictions and social distancing are found to have significant impacts on controlling the development of epidemics in many countries. In particular, we have quantified the decrease in reproduction number $R_t$ resulting from the intervention. As an illustration shown in Fig. 1, for the Hessen state in Germany, we are able to quantify that intervention in this state reduced $R_t$ by about 1 unit compared with the counterfactual scenario of no intervention.

Our findings support the presence of large heterogeneity^23^ in the reduction of $R_t$ across different states in Germany, as shown in Fig. 2. In this country, intervention measures have led systematically to a decrease in $R_t$, with quite strong differences from state to state. Even more surprising is the time dependence, which exhibits a non monotonous and fluctuating behaviour of $R_t$ in different regions.

For most regions in different countries, because of the interventions, $R_t$ decreased, but there are some places where it increased. Because this increase is transient and constrained by the lockdown, it does not lead to a very strong explosion of new cases^23,24^. A tentative interpretation is that, as the lockdown was considered and being implemented, in a number of regions, it triggered large movements of people to relocate, hence promoting new contagions and epicenters for the epidemics to mature after the lockdown^23–25^. An additional mechanism, which underlies the Japanese policy, is the effect of closed spaces and close-contact settings within confined households, which has been shown to lead to increased infections within households, for instance^23^. But because the lockdown only allowed the new nucleii of contagion to develop locally, the number of cases did not explode. Examples of this effect can be found in Luzern and Solothurn in Switzerland, in Bremen in Germany, in Saga Ken in Japan and in Odisha in India. This effect, which has previously been described qualitatively, is given quantitative support by our systematic analysis.

The observation that lockdown led first to an increase in $R_t$ in a significant number of regions and countries is confirmed by the correlation analysis presented in Fig. 9 relating the increase in cases to changes of $R_t$ in different countries. There are many regions in each country where there were no epidemics before the lockdown. The lockdown triggered a preemptive movement of people to relocate, increasing $R_t$ after the lockdown. But the increase in $R_t$ did not increase $\Delta S$ too much due to the effect of confinement. The numerous local infectious cases could only infect their immediate relatives. In regions where the epidemic was at more advanced stages, the lockdown had the effect of decreasing $R_t$, as expected. The insets in Fig. 9 provide further support for this conclusion. The average correlations shown by the plots indicate that, where there was no epidemic, the epidemic started after the lockdown, and where there was an on-going epidemic, it came under control.

### Implications for future intervention measures

We quantify that there is not much advantage resulting from strict lockdowns. We demonstrate that behavioral changes caused by intervention measures have a considerable influence on epidemic control when compared to the strictness of lockdowns. For example, Japan and Switzerland did rather well in spite of weaker lockdowns, whereas Italy and Spain did much more poorly with stricter confinements. This is related to the effectiveness of early stage contact tracing, healthcare facility, testing options as well as effective awareness of the role of protection measures. Figure 7 exemplifies this point by showing the paradoxical results that lockdown led to an obvious strong collapse in mobility accompanied by an increase of $R_t$ in several regions in France or Italy, for instance. For the other countries, the effect of the lockdown is more as expected.

Moreover, the timing of the lockdown is very important in determining the trajectory of the epidemic. Figure 8 shows the spatial correlation of the total growth of the epidemic after the lockdown. The larger the spatial diffusion (possible during the advanced stage of the epidemic), the larger is the spatial correlation. Our analysis shows that, for Italy, Switzerland, the United States, and Japan, the spatial correlation of $\Delta S$ is significantly positive. This means that, in these countries, the epidemic started much before the lockdown date and was developing silently, as revealed by the strong spatial diffusion of the epidemic. While Switzerland and Japan contained the epidemic with effective containment policies, Italy and the United States failed to do so because the intervention was ill-adapted to the spatial developments.

When comparing the German, Swiss and US lockdowns via their mobility data, we find very similar severity levels of the confinements. However, the effectiveness of the lockdown to control the epidemic in the USA is quite low, while it is very significant in Germany^26^ and Switzerland. While Switzerland and the USA both imposed a lockdown at a rather late stage of their unfolding epidemic, the Swiss containment and awareness policy was significantly superior to that in the USA. We quantify that the epidemic has diffused to many states in the USA, as revealed by the spatial correlation in Fig. 8, even after the lockdown was implemented. This explains the failure to reduce the transmission to a large degree. For Italy and Spain, because there were a significant number of confirmed cases across different regions of Italy and Spain before the lockdown, it is difficult to determine the effectiveness of cross-region transmission control.

In most Indian states, we do not observe any significant impact of the lockdown on reducing the effective reproduction number. A possible explanation is that, in most places, there is no real epidemic and the majority of the infection cases are found in a small number of states. The complete lockdown of the entire country might have been ignorant of this very strong heterogeneity.

We also unearthed some outliers, e.g, Maharastra state in India, and Saarland in Germany, where, despite a strict lockdown (quantified in our analysis by a strong reduction in workplace activity), both the number of cases and $R_t$ exploded. This poses the questions of unobserved contagion paths, likely associated with specific events, perhaps the existence of super-spreaders, and so on.

Our analysis overall suggests that the interventions may not have been optimal in many countries and that there are probably better alternatives to complete lockdowns. One alternative is a sequential and selective lockdown approach, putting in selective quarantine based on a threshold value for the number of confirmed cases while leaving the other places more open with social distancing but not complete lockdown. One should, however, stress that this alternative intervention requires very strong testing support in order to determine with sufficient reliability the positive cases. The case of India supports the idea that the policy was too early in implementing a complete lockdown of the country for such a long time, but too late in implementing an effective quarantine of people coming from affected places. The cases of Italy, France, and other regions where confinements led to a transient increase of $R_t$ over the following 30 days also underlie the plausible importance of controlling close contacts and confined places: with the aim of doing good, confining might have worsened the transmission of the disease in a number of cases.

## Conclusion

The epidemic due to the SARS-CoV-2 virus was declared a global pandemic on March 11, 2020 by the World Health Organization (WHO). Lockdown and distantiation measures of varied levels of stringency were taken by governments across the globe with the goal to control or suppress the progression of the epidemic in their respective countries. This article has presented a framework to understand and quantify the effectiveness of these interventions.

We first evaluated the time evolution of effective reproduction numbers in various geographical regions with the help of a probabilistic contagion model. We then estimated the counterfactual evolution of this effective reproduction number to quantify the impact of various lockdown measures. We observed that most regions obtained positive results in reducing the reproduction number after implementing lockdown. However, one of the surprising results of our study is that there was a transient increase in the contagion after the lockdown in some regions. We hypothetize that this resulted in part from large geographic movements and relocation of people performed in reaction to the announced lockdown measures, hence creating new contagion epicenters for the epidemics to mature after the lockdown. However, other factors, such as the dynamics of hospital/ICU admissions, would need to be considered for a better and more conclusive interpretation. The heterogeneity in observed reproduction numbers $R_t$ in different regions in this study can be attributed to different public policies applied by local governments. Also, different healthcare supports were offered in these places, including testing options. This would also affect disease spread and count.

The methodology used in our study is based on the probabilistic contagion model developed by Bettencourt and Ribeiro^20^. There exist different methods to estimate the reproduction number $R_t$ and they differ in their accuracy with pros and cons that go beyond the scope of this paper^27^. A future direction for further improvement would be to perform systematic comparisons of how different estimation methods for $R_t$ impact our main conclusions. More important would be to improve the calibration of $R_t$, both in consistency, accuracy and time resolution. ***Further, our results are based on an estimation of a counterfactual evolution***^21^ ***of the effective reproduction number. Thus the presented results are sensitive to the limitations of the above method.*** Another limitation of our study is that, for the sake of simplicity, we only considered lockdown and distantiation measures at national levels, thus neglecting regional heterogeneities. While most local lockdown measures were close to their national level policies, more accurate dates for the regional lockdown measures would improve the results of this study.

## Supplementary Information

Supplementary Information 1.

## Data Availability

All data, code, and materials used in the analysis are available at https://bit.ly/3jMrfvn.
