# Supplementary material for: Impact of Governmental interventions on epidemic progression and workplace activity during the COVID-19 outbreak
Source: Sci Rep. 2021 Nov 9;11:21939. doi: 10.1038/s41598-021-01276-5 (PMC8578600; doi:10.1038/s41598-021-01276-5)
Supplement: Supplementary file 1 — Supplementary Information 1. [file 41598_2021_1276_MOESM1_ESM.pdf]

Impact of Governmental interventions on epidemic progression and workplace activity during the COVID-19 outbreak

Sumit Kumar Ram[1*] and Didier Sornette[1-6*]

[1] ETH Zurich, Department of Management Technology and Economics
[2] ETH Zurich, Department of Earth Sciences
[3] ETH Zurich, Department of Physics
Zurich, Switzerland
[4] Swiss Finance Institute, c/o University of Geneva
Geneva, Switzerland
[5] Institute of Risk Analysis, Prediction and Management (Risks-X)
Academy for Advanced Interdisciplinary Studies
Southern University of Science and Technology (SUSTech), Shenzhen, 518055, China
[6]Tokyo Tech World Research Hub Initiative, Institute of Innovative Research
Tokyo Institute of Technology, Tokyo, Japan

[*]To whom correspondence should be addressed; E-mail: sram@ethz.ch, dsornette@ethz.ch

Supplementary Materials

6 Supporting figures

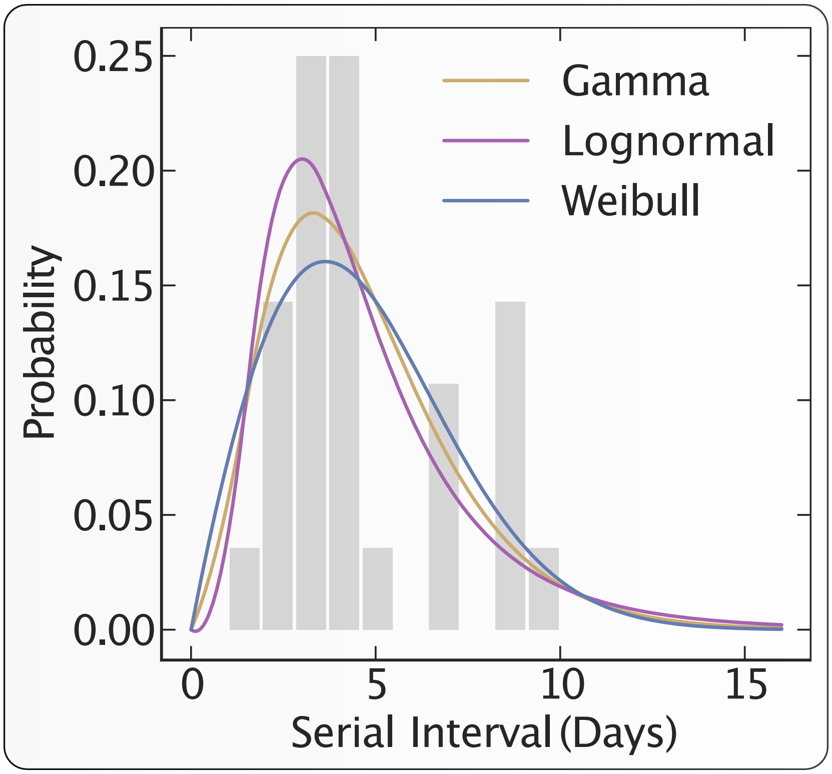

Figure S1: **Generation time distribution:** The distribution of the serial intervals are calibrated with 3 different models, i.e, Gamma, Log-normal and Weibull and using maximum likelihood estimation. We find the most suitable model for the generation time distribution by comparing the log-likelihood scores of the above models. We observe that the log-normal distribution gives the best fitting with $\mu = 4.6$ and $\sigma = 2.8$. The grey bars represents the empirical observations and the three fitted models are presented as solid lines.

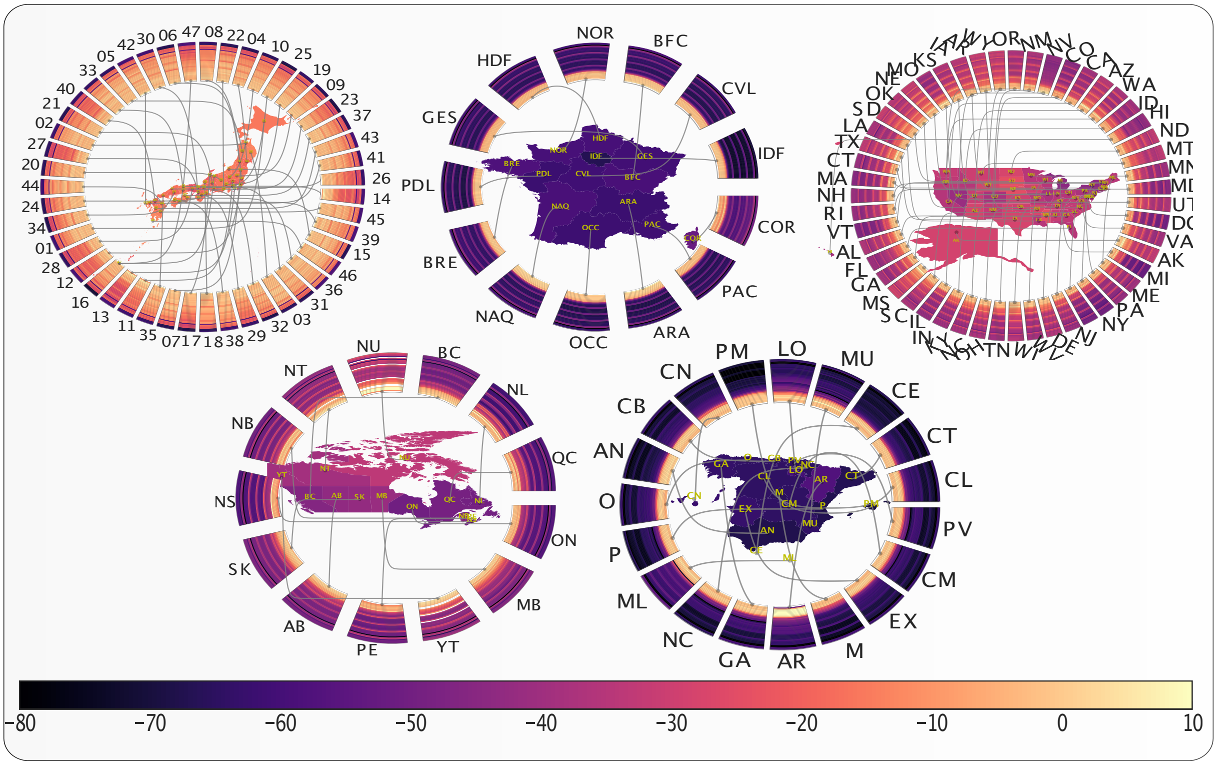

Figure S2: **Time evolution of mobility and impact of travel restriction on mobility in five countries.** Each subplot shows the time evolution of mobility and impact of travel restrictions on mobility of different top level administrative divisions of a country. The countries are, for top left to bottom right: Japan, France, USA, Canada, and Spain. The color of the regions on the map denoted by their ISO 3166-2 code represent the impact (increase or decrease of mobility) of travel restriction on that region. The radial connected wedges represent the time evolution of the mobility in the corresponding region or state for each country. The color of the strips in the wedges represent the mobility on a particular day.

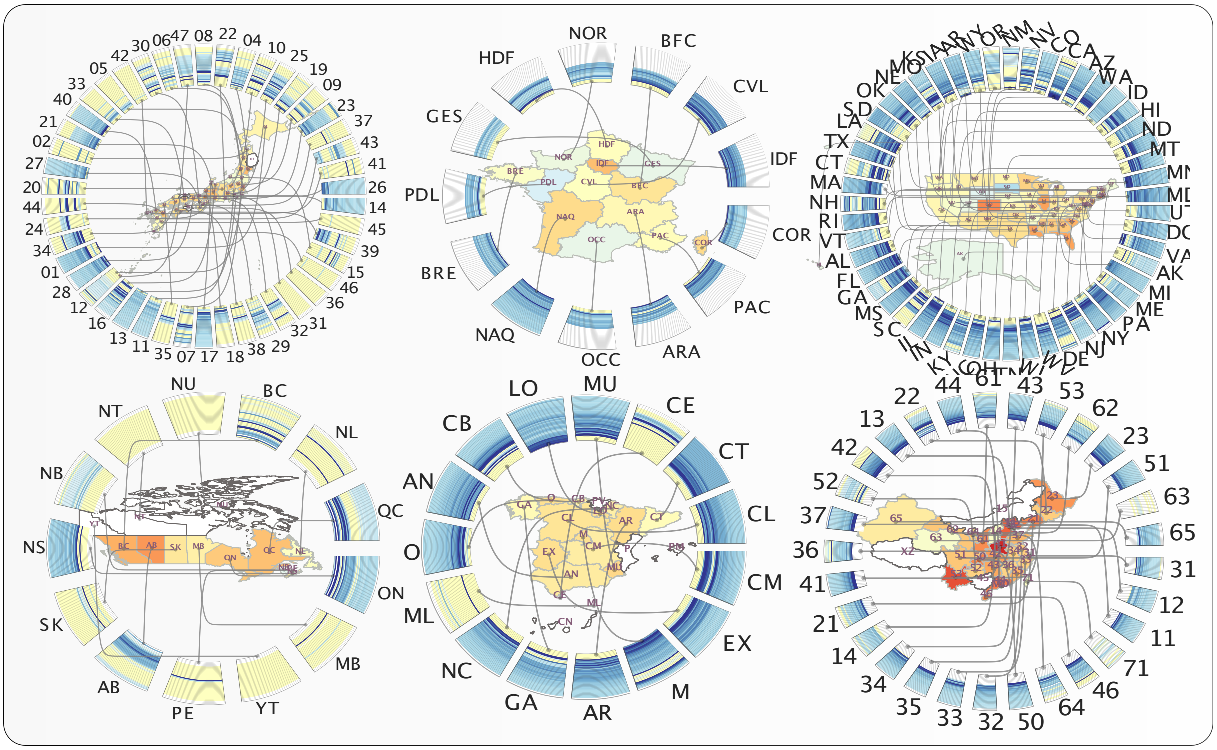

Figure S3: **Time evolution of effective reproduction number (R_t) and absolute impact of travel restrictions on R_t in six countries.** Each subplot presents time evolution of R_t and impact of interventions on R_t for different top level administrative divisions of the country. The color of the regions (name in ISO 3166-2 code notation) represents the impact (increase or decrease of R_t) of interventions on that state. The radial wedges represent the time evolution of R_t in the corresponding state, and color of the strips represent R_t on a particular day. The countries are, from top left to bottom right: Japan, France, USA, Canada, Spain and China.

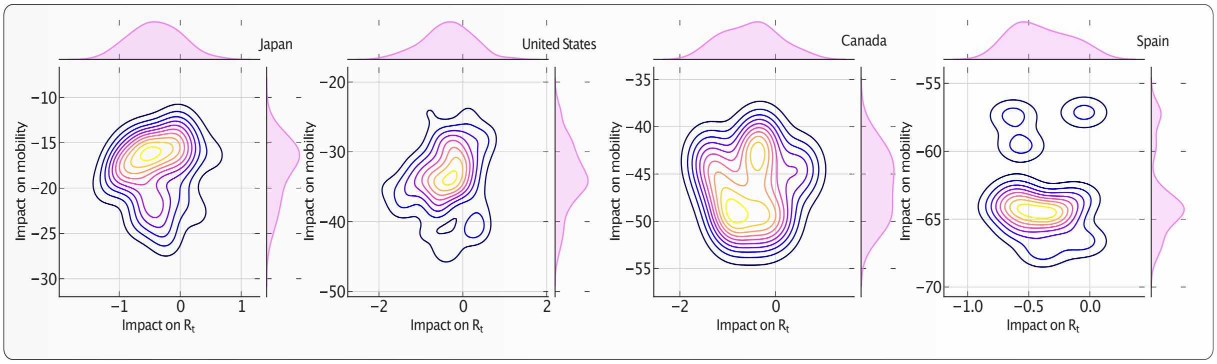

Figure S4: **Impact of travel restrictions on workplace activity and on epidemic progression (R_t) in four countries.** Each panel represents the bivariate kernel density estimation as a function of the absolute impact on workplace activity and impact on R_t in the administrative divisions of each country. The bivariate distribution is constructed over the set of regions within each country. The top and right inset of each of the four plots represent the marginal distribution of the respective variables for each country. The countries are, from left to right: Japan, USA, Canada, and Spain.

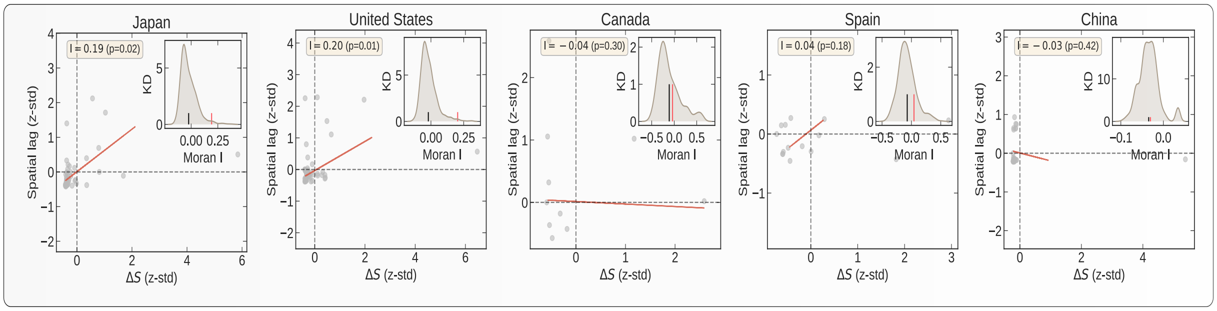

Figure S5: **Spatial auto-correlation of the total increase ΔS in number of confirmed cases during the first 30 days of intervention in five countries.** In each panel, the x-axis corresponds to the value of ΔS in a given region in a given country; the y-axis gives the average ΔS over the neighboring regions, called "spatial lag" in the caption along the y-axis. These two variables are *z-standardised* for better comparison. The inset in each panel represents the Kernel Density estimator for the distribution of the simulated *Moran's I*. The black vertical line in the inset represents the expected *Moran's I* from simulations with the null hypothesis of no spatial correlations. The red vertical line represents the value obtained from empirical data. *Moran's I* along with its p-value is given in yellow box. The countries are, from left to right: Japan, USA, Canada, Spain and China.

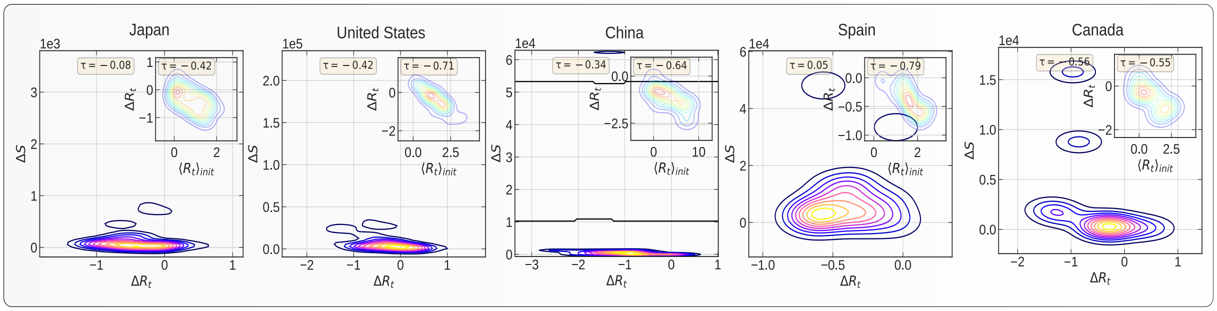

Figure S6: **Joint distribution of the total number ΔS of confirmed cases within the first 30 days of intervention and of the absolute impact of intervention on the effective reproduction number (ΔR_t) within this period.** Each panel represents the Kernel Density Estimation for the total number of confirmed cases within the first *30* days of the intervention against the impact of interventions on effective reproduction number (ΔR_t) in different administrative divisions of a country. The bivariate distribution is constructed over the set of regions within each country. The yellow box contains the Kendall τ correlation value for this joint distribution. The inset in each panel represents the variation of ΔR_t during the intervention against the average initial R_t before the intervention. The yellow box gives the corresponding Kendalls τ's. The countries are, from top left to bottom right: Japan, USA, China, Spain, and Canada.

7 Supporting Tables

7.1 Impact of interventions on effective reproduction number and growth of epidemic

Country	Administrative Division	p-value	ΔR_t	ΔS
Canada	Alberta	0	-1.39626	2061.0
Canada	British Columbia	0.027972	-1.16284	1375.0
Canada	**New Brunswick**	0.000999001	0.491501	115.0
Canada	Ontario	0.011988	-0.855158	8772.0
Canada	Quebec	0.024975	-0.967118	15783.0
Canada	Saskatchewan	0.0589411	-0.4651	303.0

Table S1: Impact of intervention on R_t and the total growth of confirmed cases in Canada. Administrative divisions with significant (confidence: 0.05) impact values are presented (the regions with positive contagion in bold face).

Country	Administrative Division	p-value	ΔR_t	ΔS
China	Anhui Province	0.03996	-0.802842	979.0
China	Beijing	0.032967	-1.26934	382.0
China	Chongqing City	0.021978	-1.56294	558.0
China	Fujian Province	0.000999001	-1.10573	283.0
China	Gansu Province	0.004995	-0.904039	89.0
China	Guangdong Province	0	-1.67239	1301.0
China	Jiangxi Province	0.00599401	-0.729367	241.0
China	Hainan Province	0.011988	-1.50345	163.0
China	Hebei Province	0.021978	-1.91644	306.0
China	Heilongjiang Province	0.00699301	-1.41766	478.0
China	Henan Province	0.013986	-2.48088	1262.0
China	Hubei Province	0	-1.33345	62218.0
China	Hunan Province	0	-1.24172	1002.0
China	Jiangsu Province	0	-0.782538	622.0
China	Jiangxi Province	0.001998	-2.23217	931.0
China	Liaoning Province	0.046953	-1.58059	118.0
China	Ningxia Hui Autonomous Region	0.002997	-0.84003	70.0
China	Shaanxi Province	0.028971	-1.48649	242.0
China	Shandong Province	0	-1.04356	743.0
China	Shanghai	0.02997	-0.619683	318.0
China	Sichuan Province	0	-0.906403	517.0
China	Yunnan Province	0.000999001	-2.06287	172.0
China	Zhejiang Province	0.002997	-0.787457	1176.0

Table S2: Impact of intervention on R_t and the total growth of confirmed cases in China. Administrative divisions with significant (confidence: 0.05) impact values are presented (the regions with positive contagion in bold face).

Country	Administrative Division	p-value	ΔR_t	ΔS
France	Nouvelle-Aquitaine	0.002997	-0.648405	3298.0

Table S3: Impact of intervention on R_t and the total growth of confirmed cases in France. Administrative divisions with significant (confidence: 0.05) impact values are presented (the regions with positive contagion in bold face).

Country	Administrative Division	p-value	ΔR_t	ΔS
Germany	**Bremen**	0	1.71413	457.0
Germany	Hamburg	0.000999001	-0.942453	3633.0
Germany	Hessen	0.038961	-0.86765	6053.0
Germany	Niedersachsen	0.003996	-0.847555	8027.0
Germany	**Saarland**	0	0.862704	2090.0
Germany	Sachsen	0.045954	-1.10722	3683.0

Table S4: Impact of intervention on R_t and the total growth of confirmed cases in Germany. Administrative divisions with significant (confidence: 0.05) impact values are presented (the regions with positive contagion in bold face).

Country	Administrative Division	p-value	ΔR_t	ΔS
India	**Maharashtra**	0.002997	0.650857	6305.0
India	**Odisha**	0	0.494264	87.0

Table S5: Impact of intervention on R_t and the total growth of confirmed cases in India. Administrative divisions with significant (confidence: 0.05) impact values are presented (the regions with positive contagion in bold face).

Country	Administrative Division	p-value	ΔR_t	ΔS
Italy	Puglia	0.026973	-0.778201	2464.0
Italy	Emilia-Romagna	0	-0.381702	16439.0
Italy	Liguria	0.037962	-0.734625	4648.0
Italy	Marche	0	-1.00811	4387.0

Table S6: Impact of intervention on R_t and the total growth of confirmed cases in Italy. Administrative divisions with significant (confidence: 0.05) impact values are presented (the regions with positive contagion in bold face).

Country	Administrative Division	p-value	ΔR_t	ΔS
Japan	Aichi Ken	0.004995	-0.553495	238.0
Japan	Akita Ken	0.03996	-0.362313	5.0
Japan	**Chiba Ken**	0.002997	0.297088	572.0
Japan	Fukui Ken	0.000999001	-0.988835	57.0
Japan	Fukuoka Ken	0.034965	-0.573188	451.0
Japan	Gifu Ken	0	-1.02953	81.0
Japan	Hyogo Ken	0.00799201	-0.721471	447.0
Japan	Ibaraki Ken	0.048951	-0.935754	90.0
Japan	Kochi Ken	0.017982	-0.676786	36.0
Japan	Kyoto Fu	0.00799201	-0.416234	196.0
Japan	Miyagi Ken	0	-1.02827	56.0
Japan	Okayama Ken	0.00899101	-0.567645	11.0
Japan	Osaka Fu	0.016983	-0.359852	1217.0
Japan	**Saga Ken**	0	0.564563	34.0
Japan	Tochigi Ken	0.0579421	-0.567938	34.0
Japan	Tokyo To	0.036963	-0.201202	3611.0
Japan	Yamagata Ken	0.01998	-0.612601	50.0
Japan	Yamaguchi Ken	0.037962	-0.4729	21.0
Japan	Yamanashi Ken	0.00999001	-0.855139	34.0

Table S7: Impact of intervention on R_t and the total growth of confirmed cases in Japan. Administrative divisions with significant (confidence: 0.05) impact values are presented (the regions with positive contagion in bold face).

Country	Administrative Division	p-value	ΔR_t	ΔS
Spain	Asturias	0.048951	-0.571728	1958.0
Spain	La Rioja	0.00899101	-0.562582	3304.0
Spain	Madrid	0.000999001	-0.568361	48108.0

Table S8: Impact of intervention on R_t and the total growth of confirmed cases in Spain. Administrative divisions with significant (confidence: 0.05) impact values are presented (the regions with positive contagion in bold face).

Country	Administrative Division	p-value	ΔR_t	ΔS
Switzerland	Appenzell Ausserrhoden	0.04995	-2.53811	68.0
Switzerland	Bern	0.016983	-1.3457	1347.0
Switzerland	Fribourg	0	-0.764614	834.0
Switzerland	Genve	0	-0.749412	3902.0
Switzerland	Graubnden	0.0599401	-0.690593	625.0
Switzerland	**Luzern**	0	0.384942	524.0
Switzerland	Neuchtel	0.001998	-0.370057	506.0
Switzerland	**Solothurn**	0	0.292663	282.0
Switzerland	St. Gallen	0.0569431	-2.59314	617.0
Switzerland	Ticino	0.00899101	-1.08246	2544.0
Switzerland	Valais	0.001998	-1.13367	1535.0
Switzerland	Vaud	0.00599401	-1.08239	4125.0
Switzerland	Zrich	0	-1.19565	2739.0

Table S9: Impact of intervention on R_t and the total growth of confirmed cases in Switzerland. Administrative divisions with significant (confidence: 0.05) impact values are presented (the regions with positive contagion in bold face).

Country	Administrative Division	p-value	ΔR_t	ΔS
United States	**Alaska**	0.0559441	0.44278	303.0
United States	Colorado	0.00599401	-1.53705	8459.0
United States	Connecticut	0.004995	-1.7927	16713.0
United States	Florida	0.004995	-1.27691	23729.0
United States	Georgia	0	-0.887848	16907.0
United States	Louisiana	0.000999001	-1.28319	22771.0
United States	Maryland	0.002997	-0.52522	11465.0
United States	Massachusetts	0	-0.700434	34896.0
United States	Michigan	0	-0.460859	26631.0
United States	Minnesota	0	-0.768463	1982.0
United States	Mississippi	0.035964	-0.80443	3743.0
United States	New Jersey	0.040959	-0.934117	77725.0
United States	Pennsylvania	0.011988	-0.584873	29256.0
United States	South Carolina	0.03996	-1.14461	3871.0
United States	**South Dakota**	0	1.08317	1400.0
United States	**Wyoming**	0.020979	0.536284	278.0

Table S10: Impact of intervention on R_t and the total growth of confirmed cases in United States. Administrative divisions with significant (confidence: 0.05) impact values are presented (the regions with positive contagion in bold face).

8 Supplemental figures

8.1 Mobility Impact

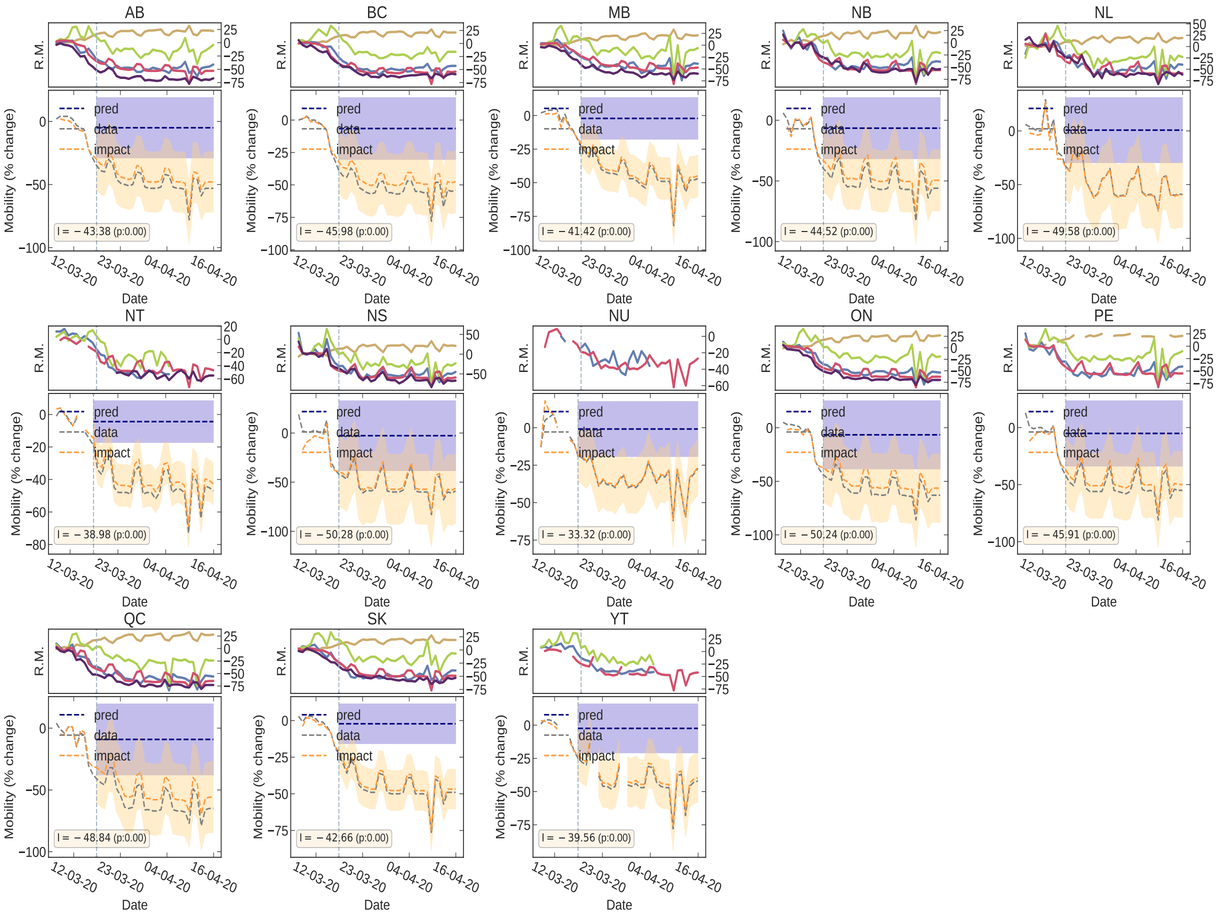

Figure S7: Impact of Governmental interventions on mobility in different administrative divisions of Canada.

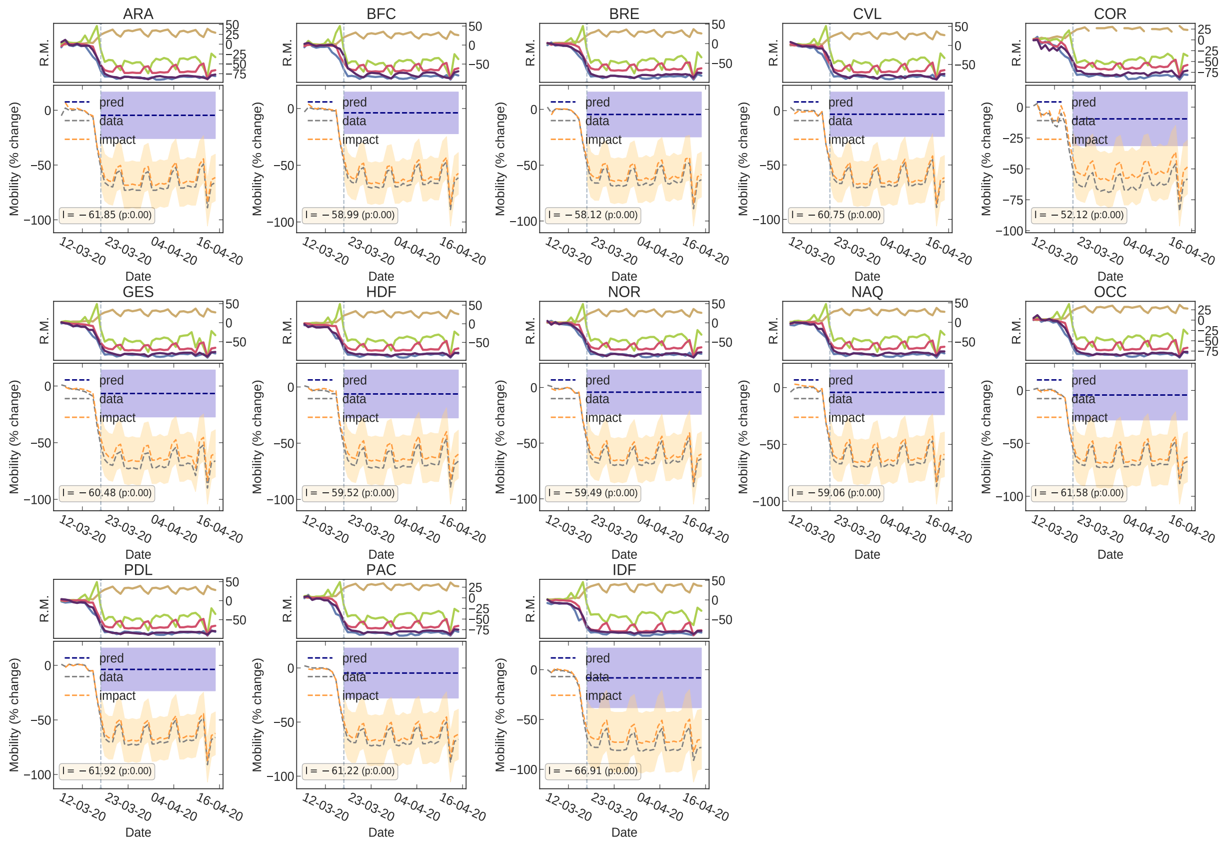

Figure S8: Impact of Governmental interventions on mobility in different administrative divisions of France.

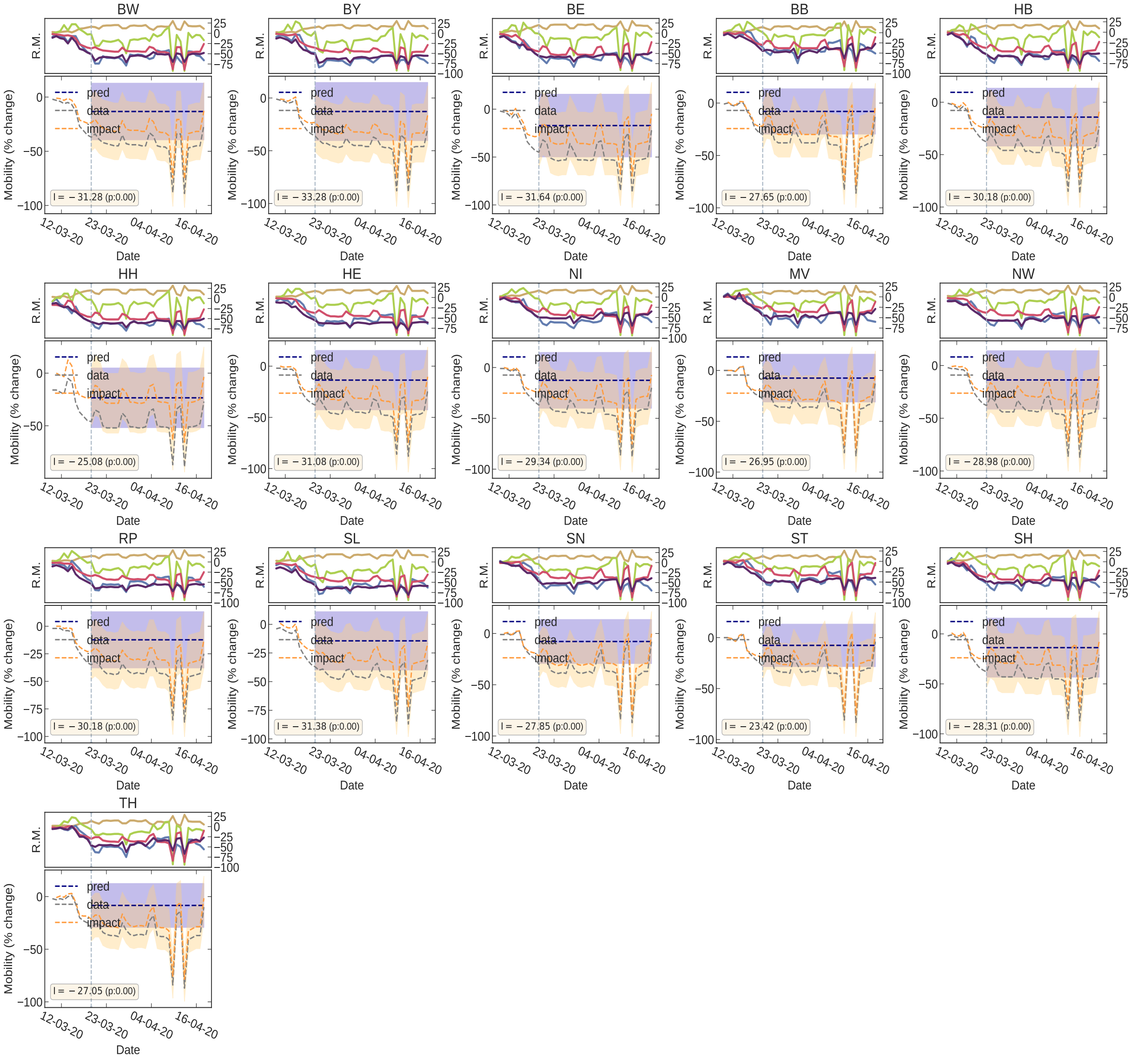

Figure S9: Impact of Governmental interventions on mobility in different administrative divisions of Germany.

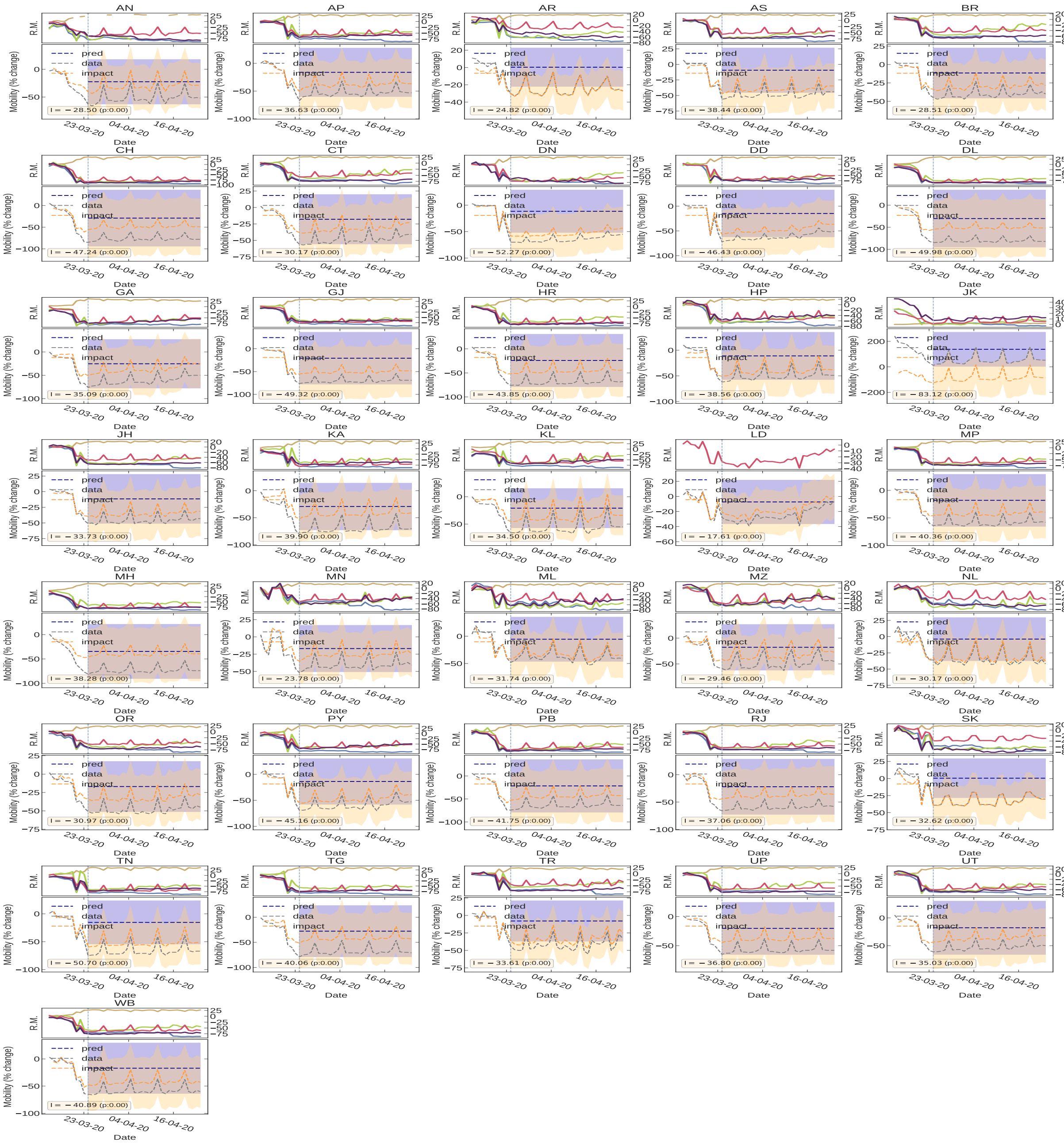

Figure S10: Impact of Governmental interventions on mobility in different administrative divisions of India.

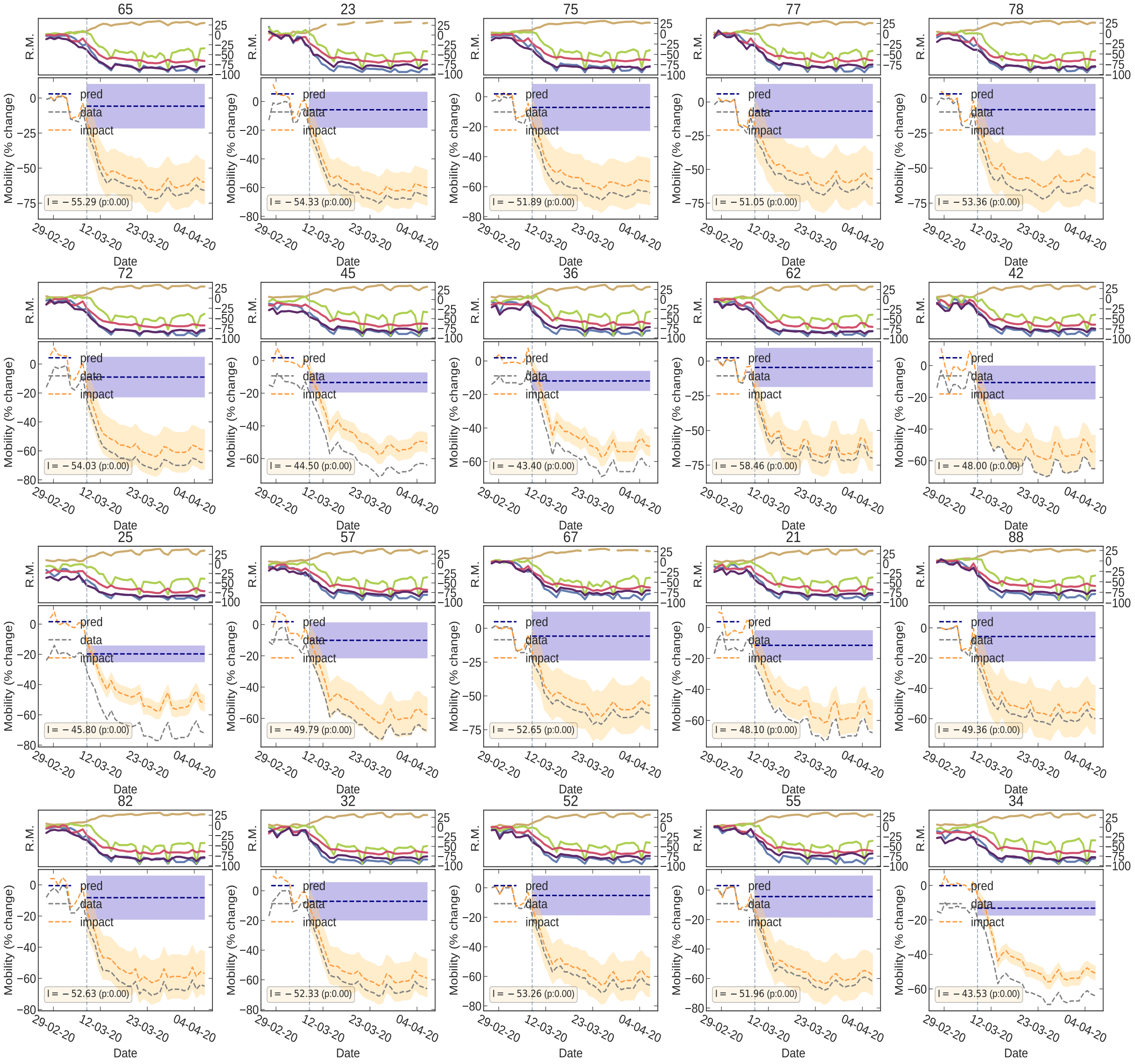

Figure S11: Impact of Governmental interventions on mobility in different administrative divisions of Italy.

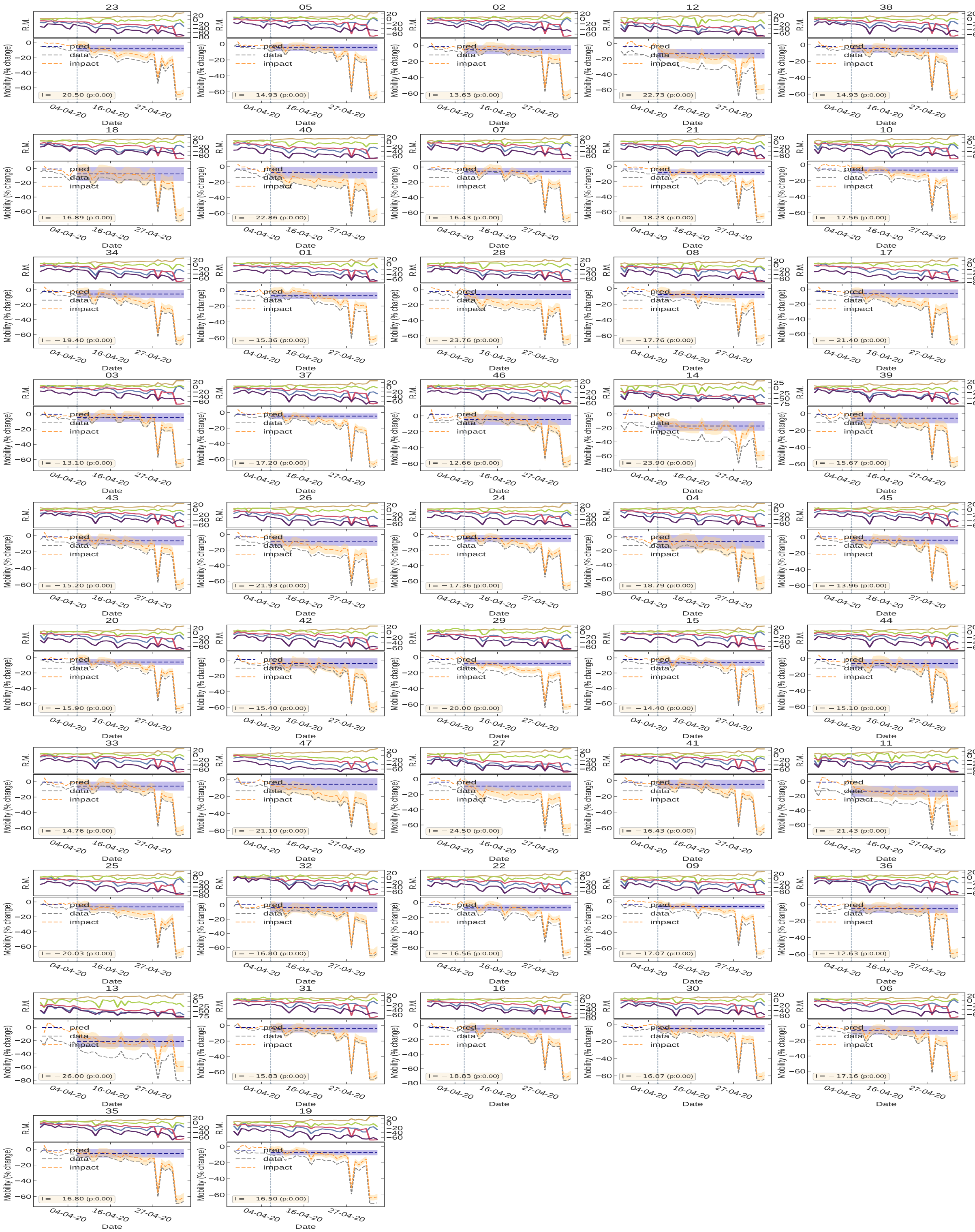

Figure S12: Impact of Governmental interventions on mobility in different administrative divisions of Japan.

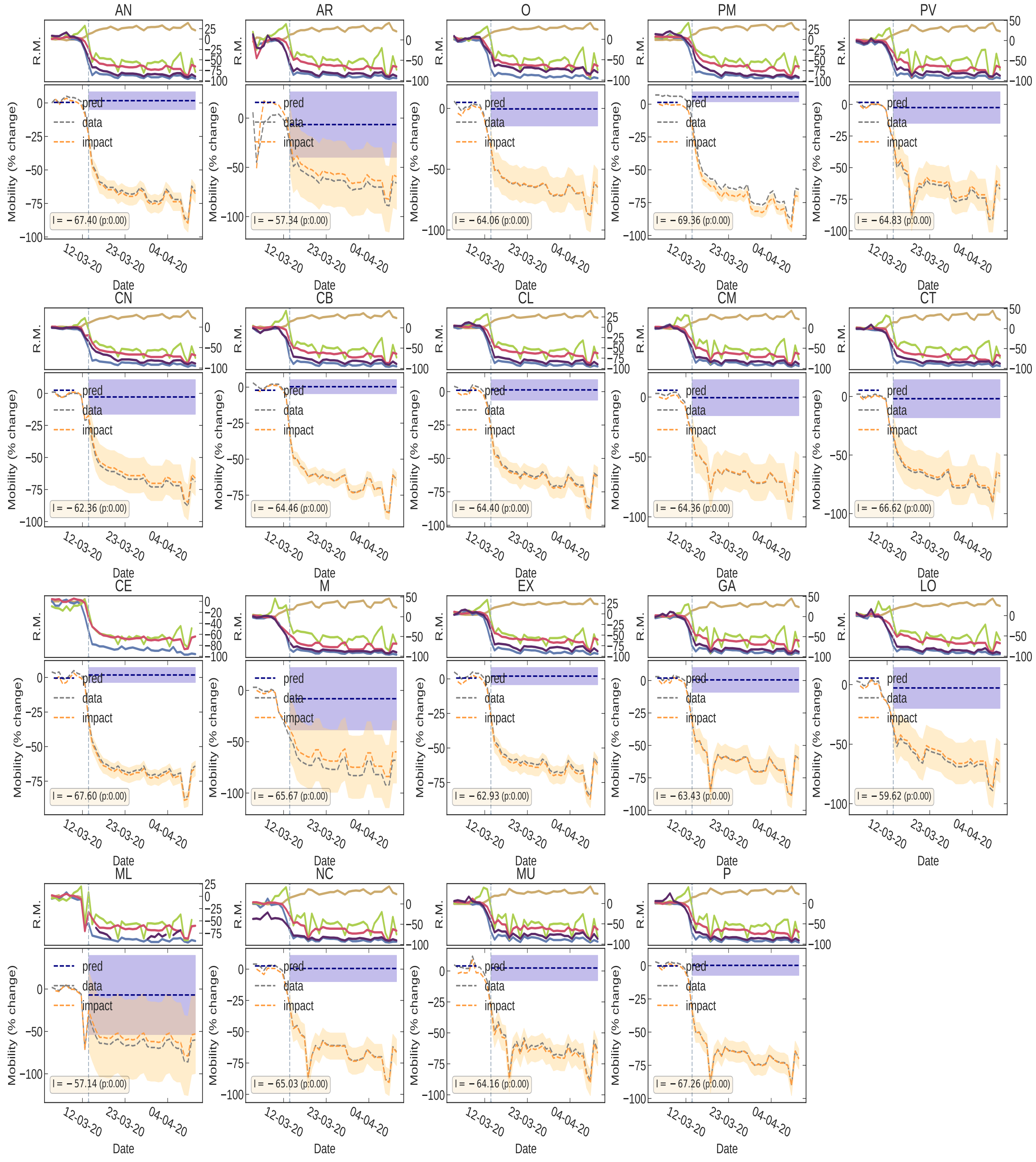

Figure S13: Impact of Governmental interventions on mobility in different administrative divisions of Spain.

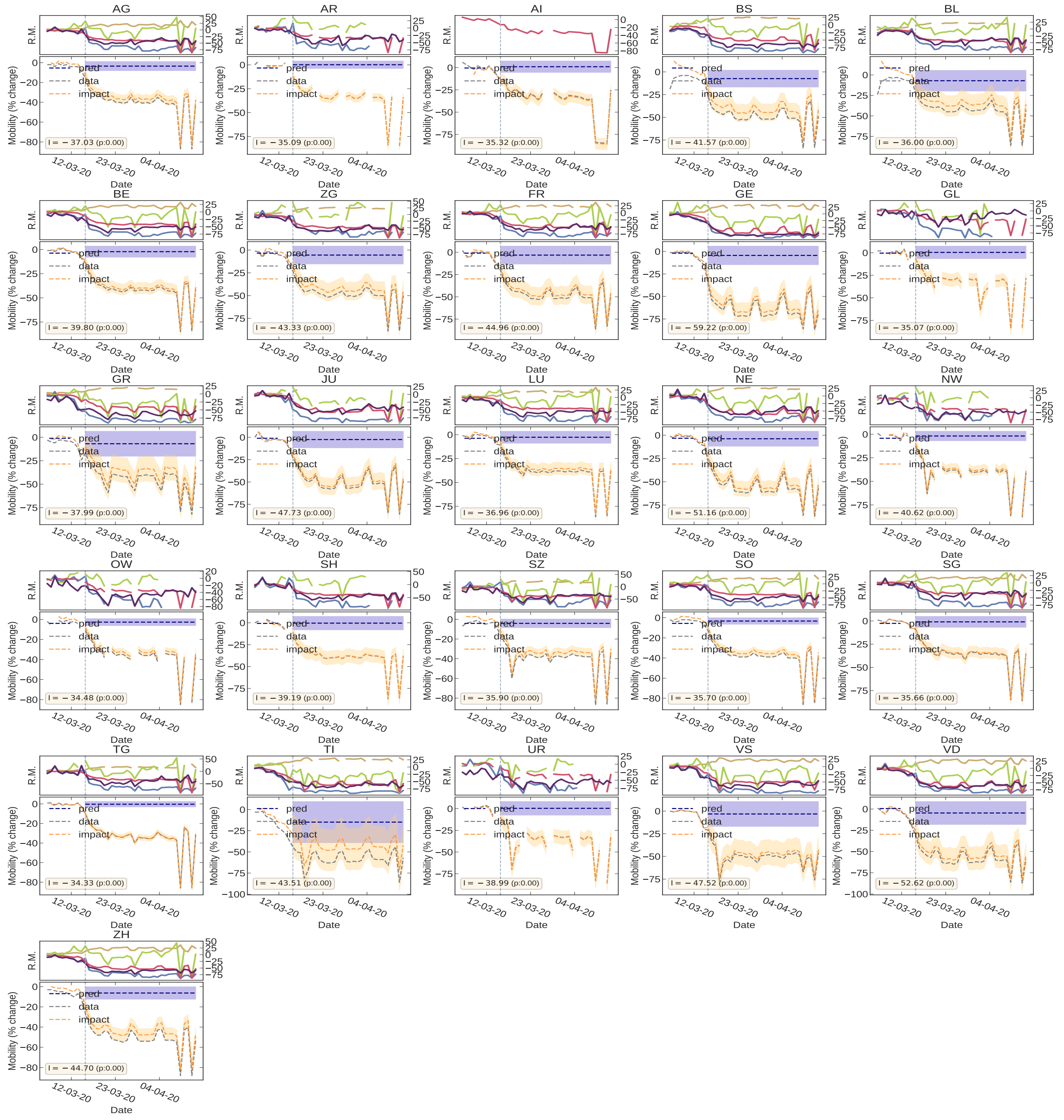

Figure S14: Impact of Governmental interventions on mobility in different administrative divisions of Switzerland.

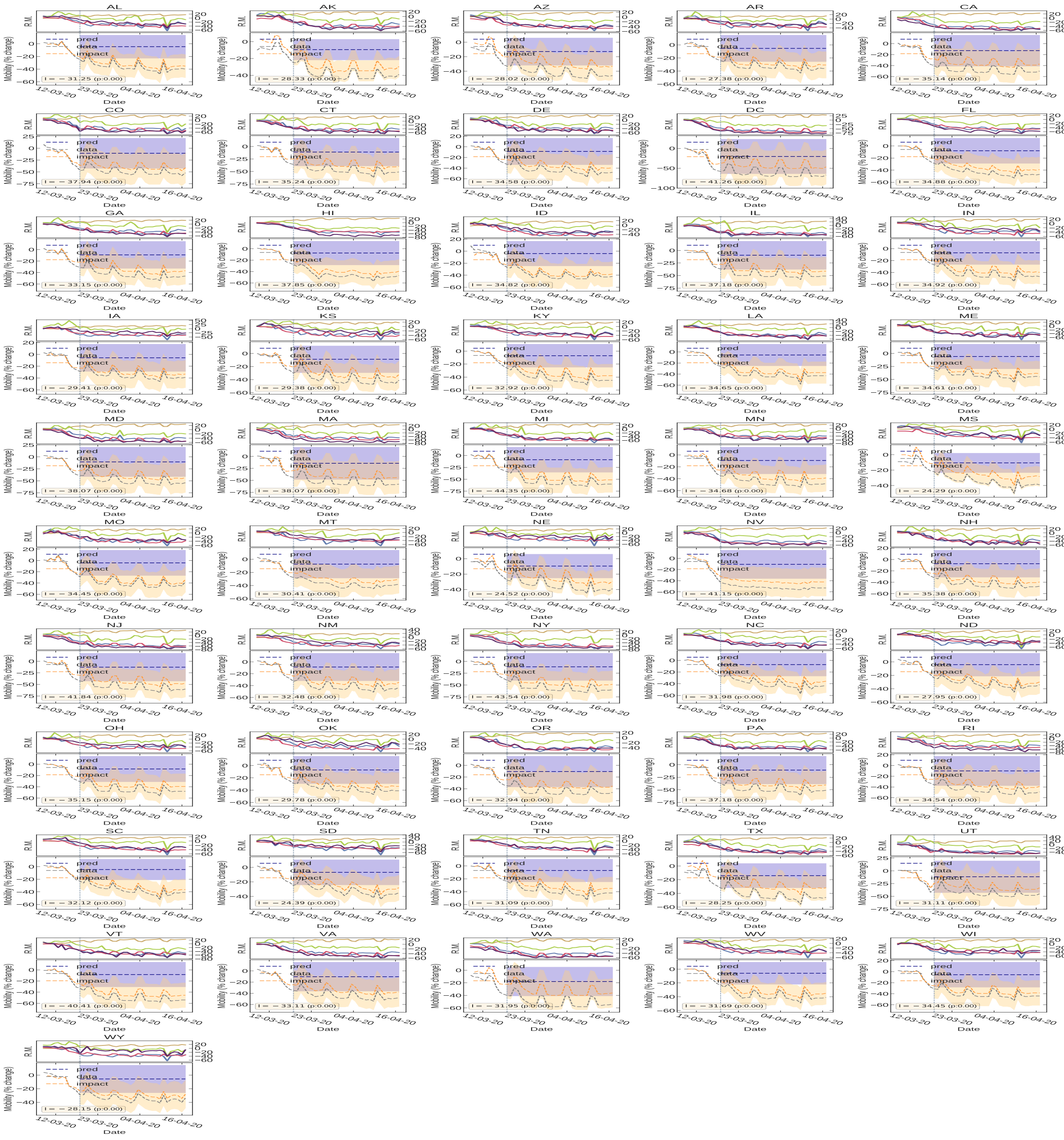

Figure S15: Impact of Governmental interventions on mobility in different administrative divisions of United States.

8.2 Epidemic Impact

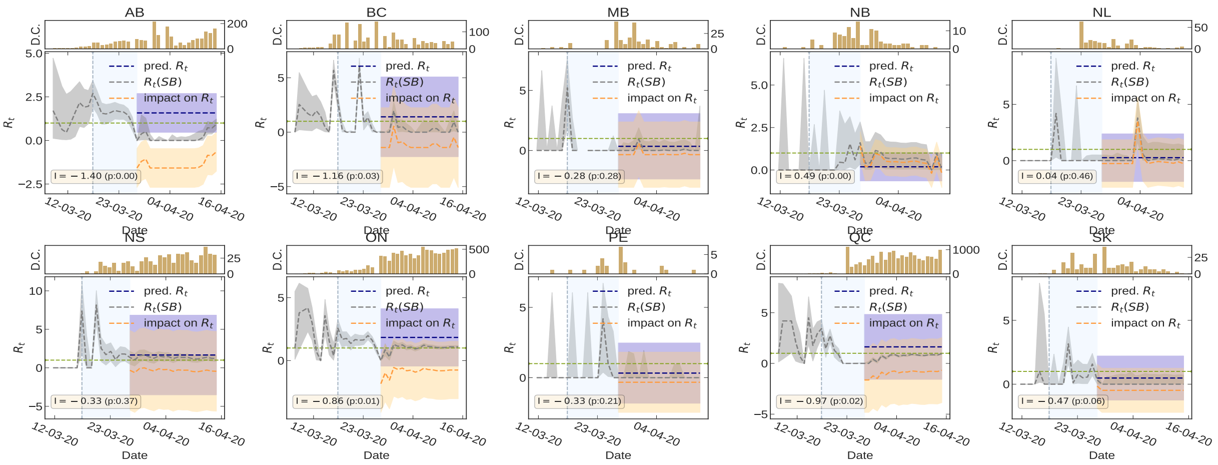

Figure S16: Impact of Governmental interventions on R_t in different administrative divisions of Canada.

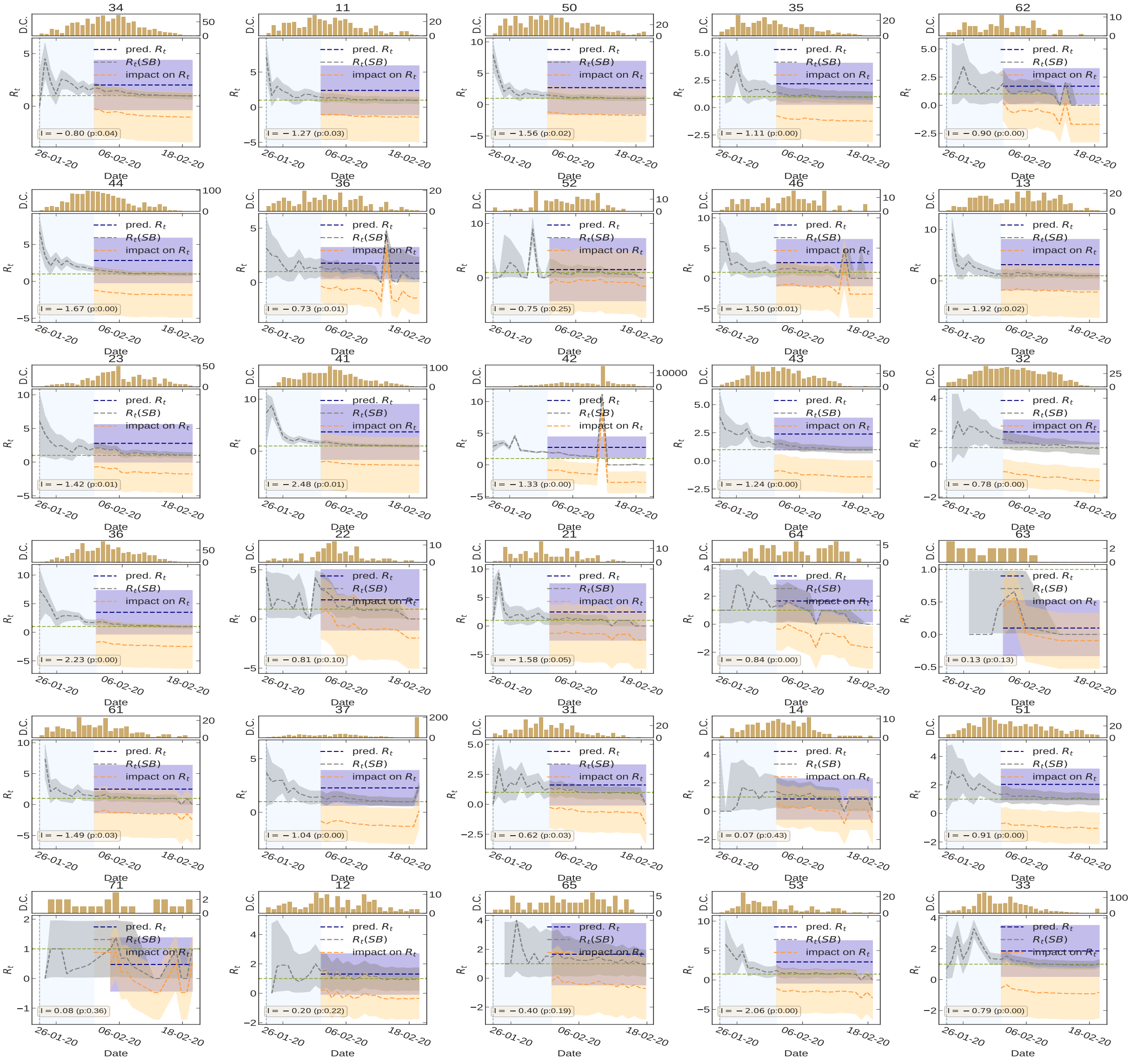

Figure S17: Impact of Governmental interventions on R_t in different administrative divisions of China.

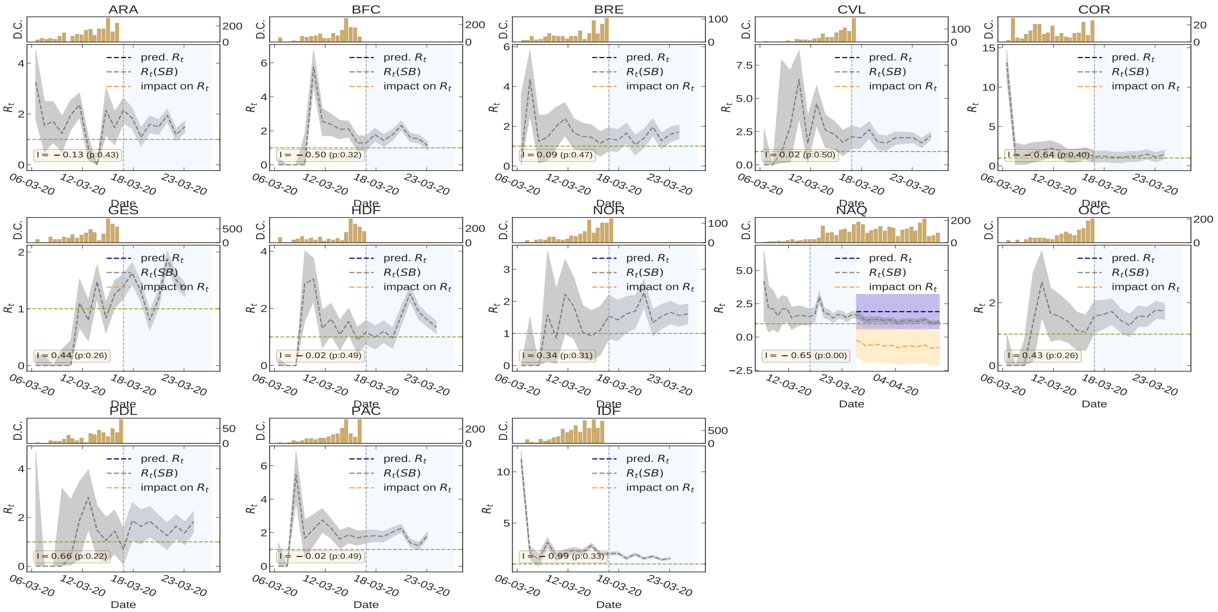

Figure S18: Impact of Governmental interventions on R_t in different administrative divisions of France.

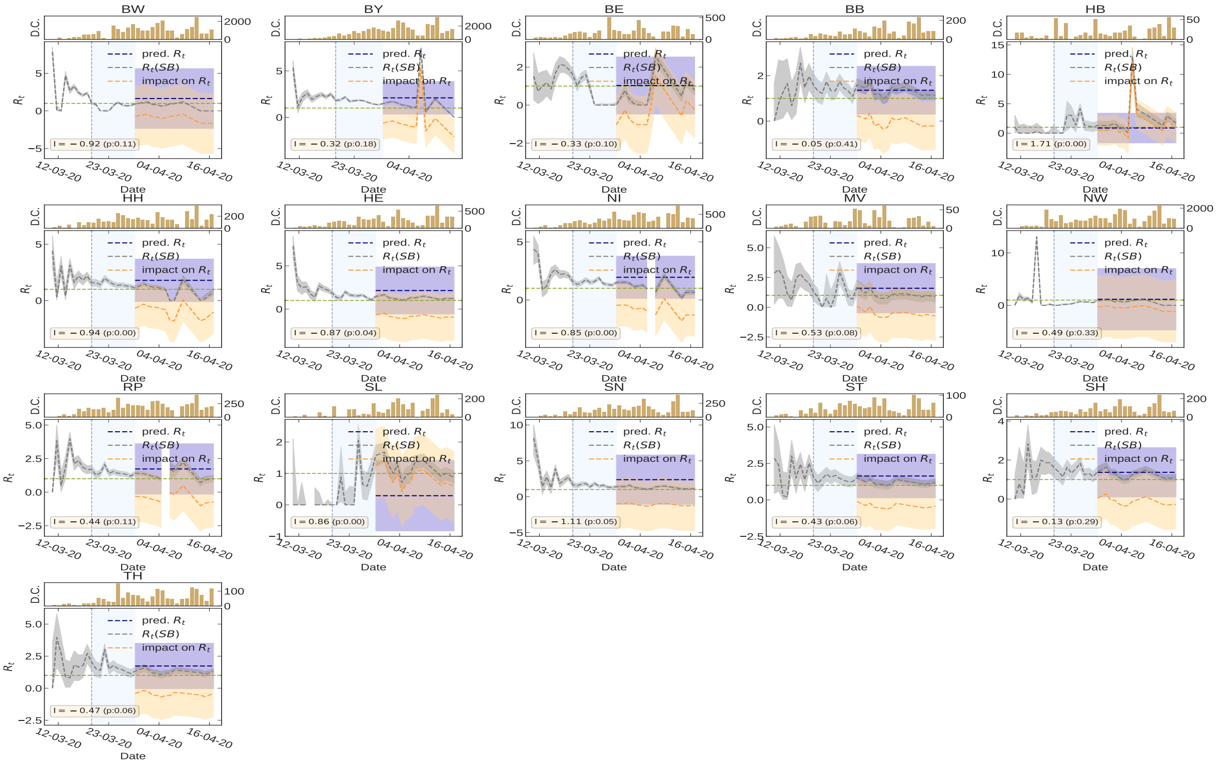

Figure S19: Impact of Governmental interventions on R_t in different administrative divisions of Germany.

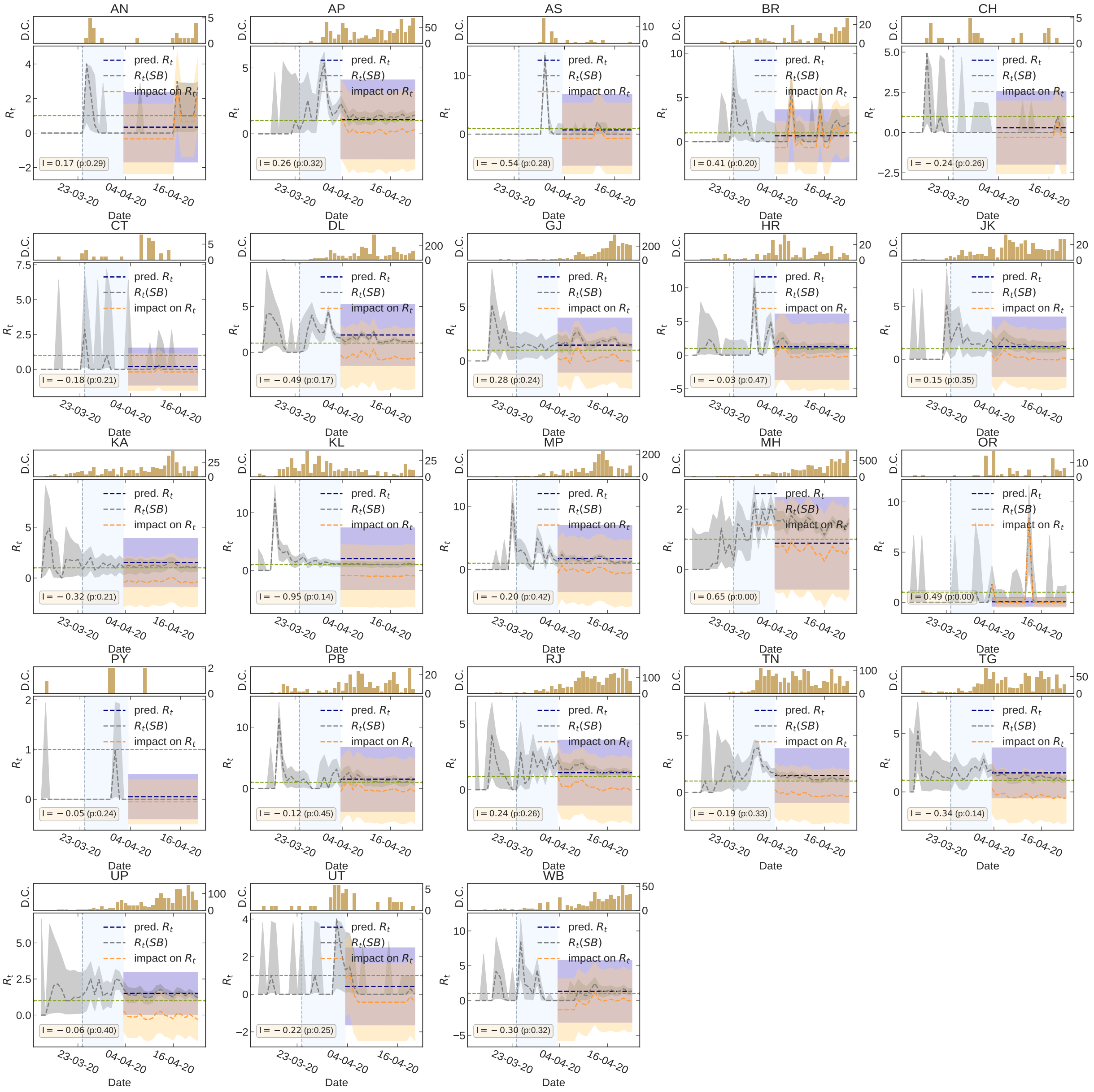

Figure S20: Impact of Governmental interventions on R_t in different administrative divisions of India.

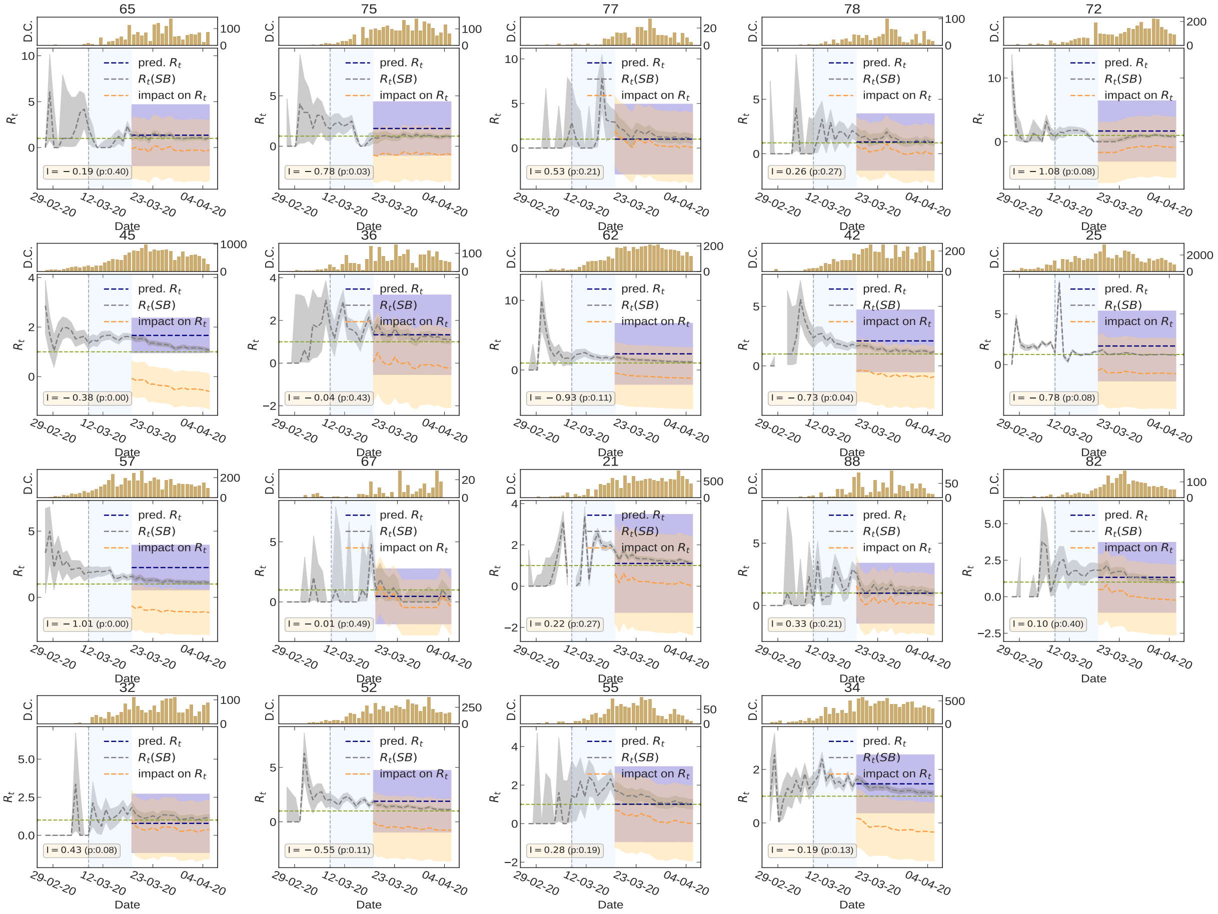

Figure S21: Impact of Governmental interventions on R_t in different administrative divisions of Italy.

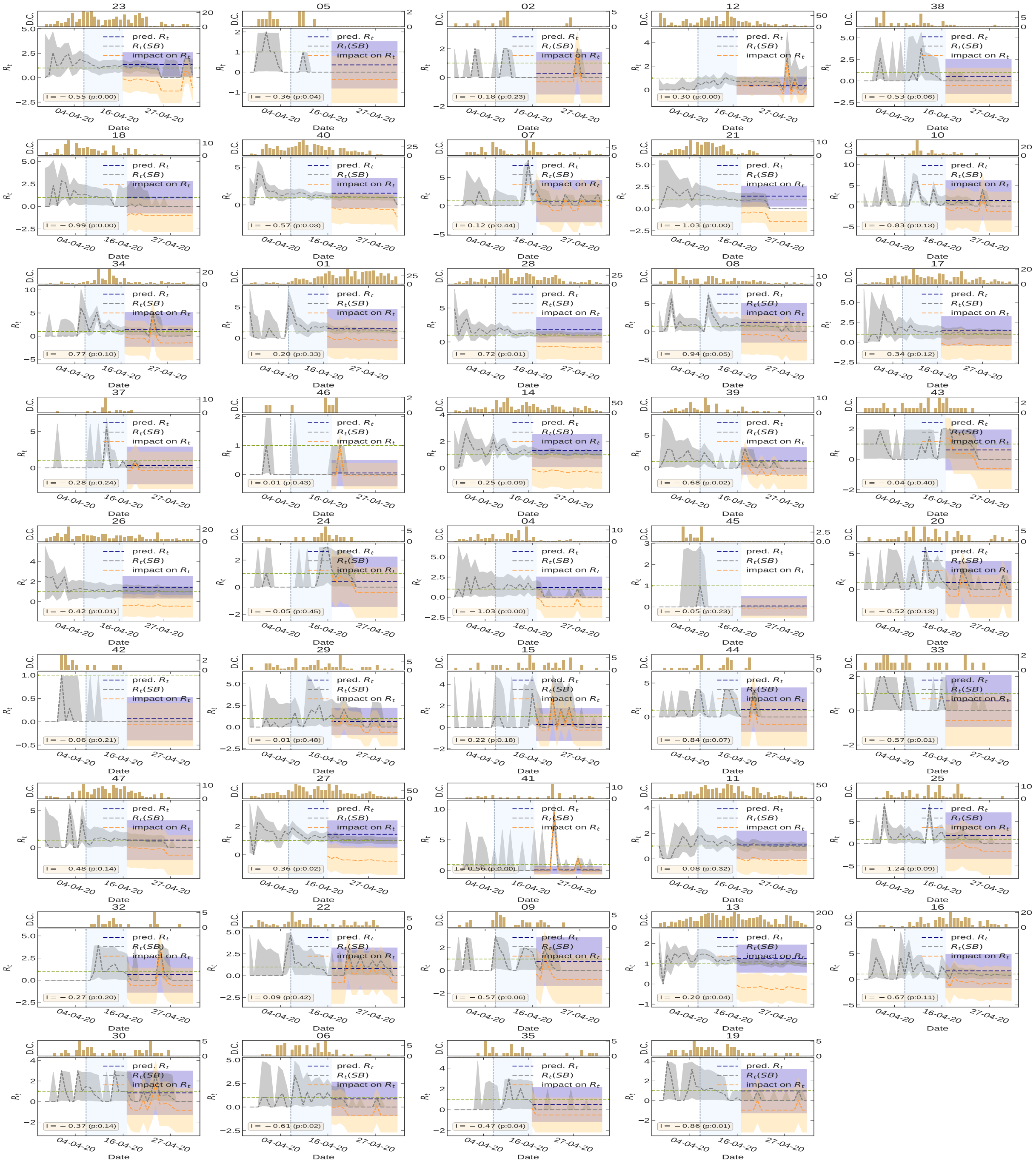

Figure S22: Impact of Governmental interventions on R_t in different administrative divisions of Japan.

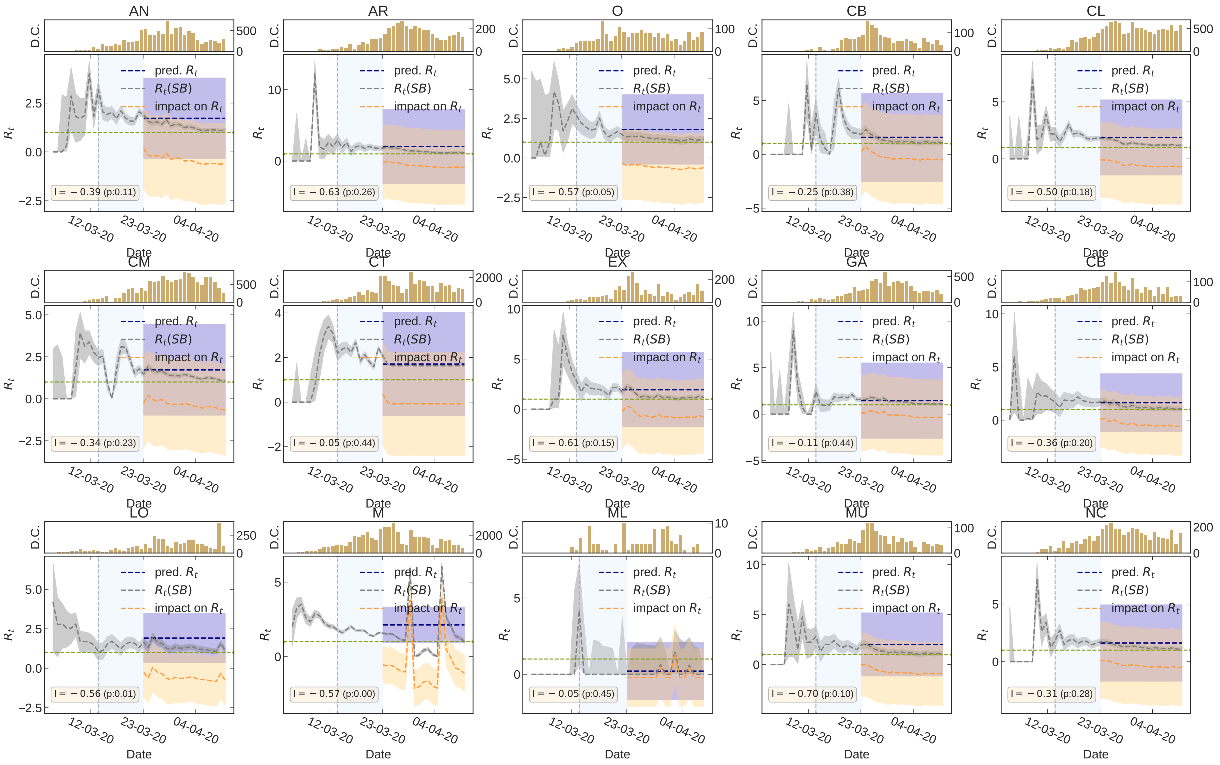

Figure S23: Impact of Governmental interventions on R_t in different administrative divisions of Spain.

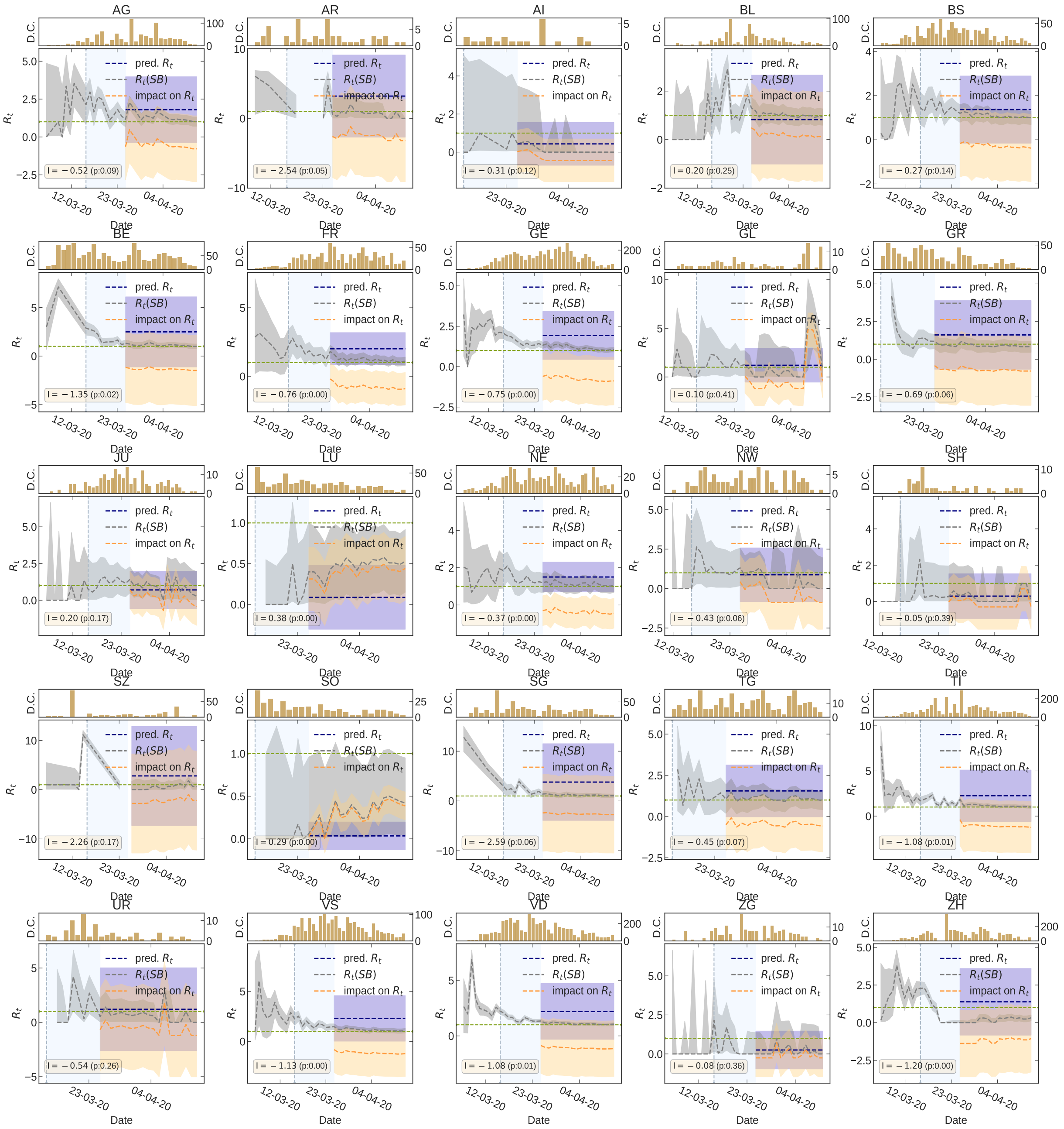

Figure S24: Impact of Governmental interventions on R_t in different administrative divisions of Switzerland.

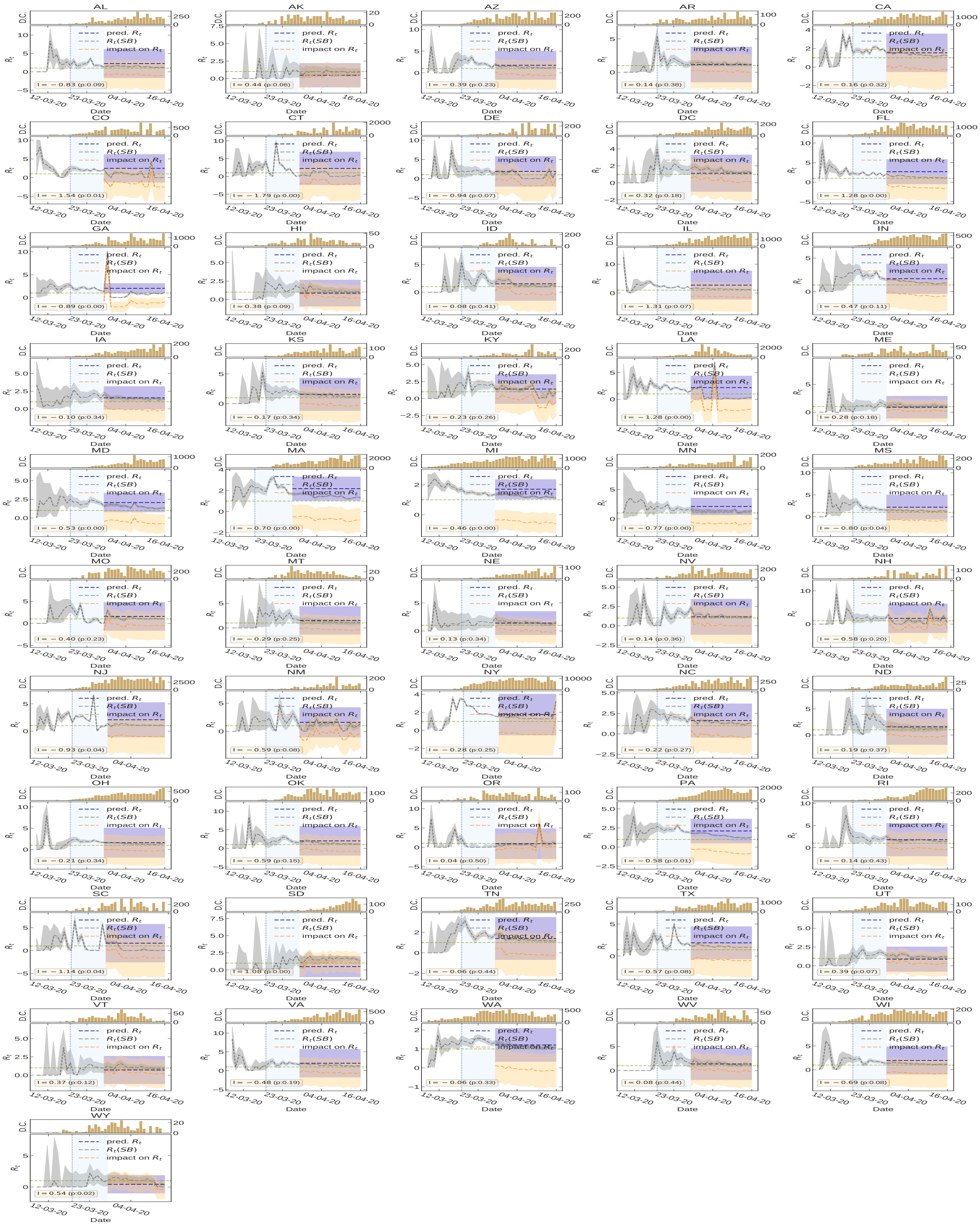

Figure S25: Impact of Governmental interventions on R_t in different administrative divisions of United States.
